# Genetic testing for diagnosing neurodevelopmental disorders and epilepsy: a systematic review and meta-analysis

**DOI:** 10.1186/s13643-025-02896-y

**Published:** 2025-07-28

**Authors:** Yu-Ming Chang, Yen-Ta Huang, Pei-Chun Lai

**Affiliations:** 1 Department of Genetic and Genomic Medicine, National Cheng Kung University Hospital, College of Medicine, National Cheng Kung University, Tainan, Taiwan; 2 Department of Pediatrics, National Cheng Kung University Hospital, College of Medicine, National Cheng Kung University, Tainan, Taiwan; 3 Department of Surgery, College of Medicine, National Cheng Kung University Hospital, National Cheng Kung University, Tainan, Taiwan; 4 Education Center, National Cheng Kung University Hospital, College of Medicine, National Cheng Kung University, 138, Sheng Li Rd., North Dist, Tainan, Taiwan

**Keywords:** Neurodevelopmental disorder, Epilepsy, Next-generation sequencing, Chromosomal microarray, Genetic test, Diagnostic yield

## Abstract

**Background:**

Identifying the genetic causes of neurodevelopmental disorders (NDDs) and epilepsy is crucial for effective treatment and genetic counseling. Our objective was to determine the diagnostic yield of chromosomal microarray (CMA) and next-generation sequencing (NGS) methods—including targeted sequencing (TS), whole-exome sequencing (WES), and whole-genome sequencing (WGS)—in individuals with NDDs or epilepsy.

**Methods:**

We systematically searched PubMed, Embase, and the Cochrane Library through August 31, 2024. Two reviewers independently screened studies and extracted data. We included studies with ≥ 10 patients (probands) diagnosed with an NDD or epilepsy who underwent CMA, TS, WES, WGS, or WES reanalysis. Methodological quality was assessed using the Newcastle–Ottawa Scale (NOS). Random-effects meta-analysis was performed to pool diagnostic yield percentages. Subgroup analyses were conducted by test modality, disorder subtype, and clinical features.

**Results:**

A total of 416 studies (124,937 participants) met inclusion criteria. Pooled analysis showed significantly higher diagnostic yields with NGS methods compared to CMA (31.1% vs 14.8% in NDD cohorts; 28.7% vs 13.3% in epilepsy cohorts). Within NGS, WES had a higher yield than targeted gene panels (35.3% vs. 23.2% for NDDs; 34.2% vs. 24.0% for epilepsy). Diagnostic yields increased over time in more recent studies. Patients with certain clinical features had particularly high yields: NDDs with dysmorphic features (54.7%), syndromic presentations (37.6%), or co-occurring epilepsy (35.6%), and epilepsy with early onset (32.3%), epileptic encephalopathy (34.7%), or drug-resistant seizures (25.4%). Quality assessment using NOS revealed that the majority of included studies were of good to very good methodological quality.

**Conclusions:**

Despite substantial between-study heterogeneity and variability in study designs that may limit the certainty of our pooled estimates, and potential publication bias, our results demonstrate that NGS-based tests—particularly WES and WGS—provide markedly higher diagnostic yields in patients with NDDs or epilepsy compared to CMA, supporting their use as first-line genetic tests. Patients with dysmorphism, syndromic NDD, early-onset or refractory epilepsy, and epileptic encephalopathy achieve above-average diagnostic yields, highlighting the value of comprehensive genetic testing in these subgroups.

**Systematic review registration:**

PROSPERO CRD42024555664.

**Supplementary Information:**

The online version contains supplementary material available at 10.1186/s13643-025-02896-y.

## Introduction

Neurodevelopmental disorders (NDDs)—including intellectual disability (ID), developmental delay (DD), and autism spectrum disorder (ASD)—and epilepsy are highly heterogeneous and multifactorial conditions that can significantly impact a patient’s quality of life and place substantial burdens on families and healthcare systems [1]. A molecular diagnosis of these disorders may alter the treatment and outcomes, as shown in a recent study reporting that 50% of patients with epilepsy who received a genetic diagnosis underwent changes in their clinical management [2]. Moreover, elucidating the underlying genetic causes can guide more personalized management, including selecting antiseizure medications tailored to specific genetic etiologies and avoiding those known to exacerbate particular syndromes. For instance, individuals with Dravet syndrome (often caused by SCN1A variants) may experience seizure aggravation with sodium channel–blocking drugs and benefit from treatments such as stiripentol or fenfluramine, whereas patients with SCN2A-related epilepsies may respond more favorably to different medication regimens [2]. In addition, recognizing specific molecular diagnoses can inform lifestyle adjustments (e.g., temperature regulation in Dravet syndrome) and provide anticipatory guidance for commonly associated comorbidities, ultimately improving overall care and outcomes [3].

NDDs and epilepsy can be caused by a variety of genetic variants, including chromosomal rearrangements, copy number variants (CNVs), small insertions or deletions (indels), or single-nucleotide variants. Karyotyping, chromosomal microarray (CMA), and a variety of sequencing technologies can be used to identify the genetic cause of such disorders, with each type of test having different strengths, limitations, and costs [4]. Hence, choosing the appropriate genetic test—based on factors such as suspected variant type, resolution, turnaround time, and cost—is paramount to making a correct molecular diagnosis.

CMA, a powerful tool for detecting CNVs, has been the first-tier genetic test for individuals with ID/DD and ASD for the last 15 years. While this test provides a higher diagnostic yield (15–20%) than G-banded karyotyping [5], its resolution is limited for the identification of SNVs and small indels. This shortcoming may be compensated by next-generation sequencing (NGS) technologies such as targeted sequencing (TS), whole-exome sequencing (WES), and whole-genome sequencing (WGS). TS focuses on sequencing specific areas or genes of interest in the genome, whereas WES sequences the coding regions of all exons. WGS more comprehensively encompasses the entire genome, covering both coding and noncoding regions, and better detects CNVs and structural variants than does WES or TS [6].

After a consensus statement published in 2019, exome sequencing is now considered a first-tier clinical diagnostic test for individuals with NDDs, given that it has a higher diagnostic yield (36%) than does CMA [7]. A meta-analysis of 103 studies published in 2020 reports that clinical sequencing has a diagnostic yield of 28% for intellectual disability, 17% for ASD, and 24% for epilepsy, supporting the use of sequencing as a first-tier test for NDDs and epilepsy [8]. Subsequently, many more studies reporting the diagnostic yield of sequencing methods have been published, especially with reanalysis and WGS. An updated meta-analysis is warranted to analyze data from these recent studies. Furthermore, the continued evolution of variant interpretation guidelines and reference databases suggests that periodic reanalysis of prior genomic data may yield additional diagnoses, making it timely to re-evaluate the diagnostic yield of these methods [9]. Recommendations based on various types of NDD and epilepsy are also needed to provide clinicians with more robust evidence. Therefore, a large-scale, up-to-date systematic review and meta-analysis incorporating increasingly widespread methods—such as WGS and reanalysis—is warranted to provide a more comprehensive understanding of the genetic diagnostic yield in NDDs and epilepsy.

## Methods

### Search strategy

**Fig. 1:**
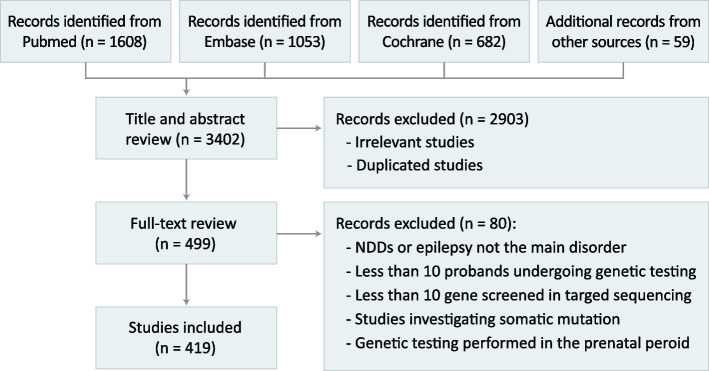
The searching, screening, and selection process

This systematic review and meta-analysis followed the Preferred Reporting Items for Systematic Reviews and Meta-Analysis (PRISMA) reporting guidelines and considered all studies available in PubMed, Embase, and Cochrane Library through August 31, 2024. Search terms used included disorder-specific terms (neurodevelopmental disorder, intellectual disability, mental retardation, developmental delay, autism, autistic spectrum disorder, epilepsy, epileptic encephalopathy, and seizure), testing method terms (chromosomal, oligonucleotide, DNA or gene combined with array, microarray, microchip, or chip, comparative genome hybridization, next-generation sequencing, exome sequencing, and genome sequencing), and other content-related terms (cohort, diagnostic yield, diagnostic test, and clinical practice). The study protocol was preregistered on PROSPERO [10]. Detailed search strategies are listed in Supplementary Table 1. Additional studies were identified through listed references. The searching, screening, and selection process is summarized in Fig. 1.

### Eligibility criteria and selection process

Studies eligible for inclusion were those with at least 10 probands with NDD or epilepsy who underwent either CMA, TS, WES, WGS, or reanalysis of sequencing data. Excluded studies were those that tested less than 10 genes, had fewer than 10 probands, did not have NDD or epilepsy as the main disorder, investigated somatic mutations, with genetic tests performed during prenatal periods, and duplicated studies. The search strategy was developed in consultation with an experienced librarian who provided guidance on keyword selection and search methodology. Two reviewers (Yu-Ming Chang and Pei-Chun Lai) collaboratively reviewed titles, abstracts, and full texts for eligibility according to the predetermined criteria, working together to reach consensus on study selection.

### Data collection and analysis

From the qualifying studies, two reviewers independently extracted the number of probands with a positive test result and the total number of probands who received the test. A positive result was defined as the detection of a pathogenic or likely pathogenic variant, a variant of unknown significance (VUS) with a strong clinical match, or a variant that was considered causative by the respective study author. Variant pathogenicity was classified according to the American College of Medical Genetics and Genomics (ACMG) system. Any discrepancies were resolved by consensus of the reviewers.

Patients in the qualifying studies were divided into subgroups for subsequent analysis. NDD was divided into ID/DD and ASD. The ID/DD subgroup was further divided according to severity (mild, moderate, or severe/profound ID) and associated features (ID/DD alone, ID/DD with ASD, ID/DD with epilepsy, ID/DD with dysmorphism, or syndromic ID/DD). Patients with epilepsy were grouped according to age at onset (before 2 years of age or later in childhood), seizure type (focal or generalized epilepsy), and other features (drug-resistant epilepsy or epileptic encephalopathy). To assess the diagnostic yield according to date, we also grouped the studies according to the published year (2019 and earlier or 2020 and after). Subgroups with at least three studies were analyzed. All subgroup cohorts are detailed in Tables 1 and 2.

**Table 1:** Diagnostic yield of chromosomal microarray and sequencing among patients with neurodevelopmental disorders and epilepsy

Group	Chromosomal microarray		Target sequencing (TS)		Whole-exome sequencing (WES)		TS or WES		Whole-genome sequencing	
	No. of cohorts/individuals	Diagnostic yield (95% CI)	No. of cohorts/individuals	Diagnostic yield (95% CI)	No. of cohorts/individuals	Diagnostic yield (95% CI)	No. of cohorts/individuals	Diagnostic yield (95% CI)	No. of cohorts/individuals	Diagnostic yield (95% CI)
Neurodevelopmental disorder	151/69,852	14.8% (13.4–16.2%)	48/6481	23.2% (20.2–26.6%)	94/13,386	35.3% (31.5–39.3%)	140/20,407	31.1% (28.3–34.2%)	18/3844	30.5% (22.8–39.4%)
By disorder										
ID/DD	101/41,187	16.9% (15.4–18.6%)	36/4854	24.3% (20.7–28.4%)	57/7308	40.0% (35.3–45.0%)	93/12,720	34.0% (30.5–37.7%)	12/1423	33.9% (28.6–39.6%)
ASD	66/23,640	10.3% (8.7–12.2%)	17/1839	16.9% (13.2–21.3%)	21/3375	20.5% (13.7–29.5%)	36/5155	17.8% (13.8–22.8%)	5/2081	12.8% (5.7–26.0%)
By year										
2019 and earlier	95/41,763	15.2% (13.5–17.0%)	25/3757	19.6% (16.0–23.8%)	24/2065	33.4% (27.1–40.4%)	50/6107	25.9% (22.0–30.1%)	5/414	38.3% (28.0–50.0%)
2020 and after	56/28,089	14.1% (12.0–16.5%)	23/2724	27.6% (23.1–32.6%)	70/11,321	36.0% (31.3–40.9%)	90/14,300	34.3% (30.6–38.3%)	13/3430	27.3% (18.8–37.8%)
Epilepsy	53/7114	13.3% (11.2–15.7%)	97/28,518	24.0% (21.5–26.7%)	93/7963	34.2% (30.9–37.6%)	173/36,533	28.7% (26.4–31.0%)	14/1313	39.1% (32.4–46.2%)
By year										
2019 and earlier	35/4210	14.0% (11.0–17.5%)	57/17,056	22.7% (19.3–26.6%)	32/2696	33.0% (28.3–38.1%)	78/19,618	25.7% (22.7–29.1%)	3/231	58.0% (14.4–92.1%)
2020 and after	18/2904	12.3% (9.8–15.4%)	40/11,462	25.6% (22.1–29.4%)	61/5267	34.8% (30.5–39.4%)	95/16,915	31.2% (28.1–34.5%)	11/1082	38.9% (31.2–47.2%)

**Table 2:** Diagnostic yield of chromosomal microarray and targeted/exome sequencing among subgroups

Subgroup	CMA		TS/WES		*P* value (for TS/WES)
	No. of cohorts/No. of individuals	Diagnostic yield (95% CI)	No. of cohorts/No. of individuals	Diagnostic yield (95% CI)	
ID (by severity)					(Compared with mild ID)
Mild	12/919	19.4% (15.8–23.5%)	7/188	32.1% (22.7–43.2%)	
Moderate	9/637	16.7% (12.3–22.1%)	6/177	40.4% (22.5–61.1%)	0.43
Severe/Profound	9/462	18.8% (15.4–22.6%)	7/340	44.7% (32.0–58.1%)	0.20
ID/DD (by associated features)					(Compared with isolated ID/DD)
ID/DD alone	5/3982	16.7% (10.6–25.3%)	11/788	24.1% (17.1–32.8%)	
With ASD	23/5160	13.8% (10.6–17.8%)	12/642	31.1% (19.9–45.0%)	0.52
With epilepsy	22/2268	18.4% (14.5–23.0%)	57/5533	35.6% (31.8–39.6%)	0.033*
With dysmorphism	18/2458	28.8% (22.7–35.8%)	8/425	54.7% (43.0–65.9%)	0.003*
Syndromic presentation	16/5466	28.1% (22.5–34.6%)	17/2318	37.6% (32.0–43.6%)	0.019*
Epilepsy (by associated features)					(Compared with isolated epilepsy)
Epilepsy alone	NA**		6/752	15.2% (11.2–20.3%)	
Drug-resistant epilepsy	5/244	12.0% (7.4–19.0%)	23/2233	25.4% (20.4–31.2%)	0.043*
Epileptic encephalopathy	15/1000	11.7% (8.5–16.0%)	58/4409	34.7% (31.8–37.7%)	0.0003*
West syndrome	10/573	10.7% (7.4–15.1%)	22/1280	27.7% (22.9–33.1%)	0.031*
Epilepsy (by onset age)					(Compared with onset after childhood)
Onset earlier than 2 years of age	7/375	15.4% (8.3–26.6%)	44/4570	32.9% (29.1–36.9%)	0.000013*
Onset later than 2 years of age	3/316	13.0% (8.4–19.6%)	18/1962	15.6% (11.8–20.4%)	
Epilepsy (by seizure type)					(Compared with generalized epilepsy
Focal epilepsy	4/314	10.1% (7.2–14.1%)	21/1793	21.3% (13.1–32.7%)	0.89
Generalized epilepsy	4/121	23.9% (16.9–32.7%)	19/1279	26.8% (17.9–38.2%)	

*p < 0.05

**Fewer than three studies identified

### Statistical analysis

The overall diagnostic yield of each cohort was evaluated using a random-effects model using the meta package in R (version 4.2.2). Logit transformation was applied for meta-analysis of single proportions, and the pooled diagnostic yield and 95% confidence interval were calculated. Plots were created using the ggplot2 package. Inter-study heterogeneity was quantified as the *I*^2^ statistic. We applied the nonparametric Wilcoxon rank-sum test to compare outcomes between two independent groups of studies due to the nonnormal (skewed) distribution of diagnostic yield values across studies and small sample sizes in some subgroups. This test is appropriate for comparing distributions and median values without assuming normality, making it suitable for evaluating differences in diagnostic yield between groups utilizing different sequencing modalities. The data were analyzed in September 2024. To visualize changes in the diagnostic yield over time, we plotted the diagnostic yield of individual studies with Freeman-Tukey double arcsine transformation versus the year of publication and added a regression line to depict the trend of the change in yield [11].

### Quality assessment

The methodological quality of included studies was assessed using the Newcastle-Ottawa Scale (NOS) adapted for cross-sectional studies [12]. Although our inclusion criteria encompassed various study designs beyond cross-sectional studies, we included all studies reporting diagnostic yield rates of genetic testing in epilepsy or neurodevelopmental delay. The decision to utilize the NOS for cross-sectional studies was based on the observation that most studies reporting diagnostic yield rates predominantly followed a cross-sectional design, which represents a straightforward approach to presenting diagnostic outcomes. Two reviewers (Pei-Chun Lai and Yen-Ta Huang) evaluated each study according to the NOS criteria. Based on team consensus, studies with sample sizes of 200 or more participants were awarded one point in the sample size domain. For comparability assessment, studies that performed meta-regression analysis were awarded two points, while those conducting subgroup or stratification analyses received one point. Studies were categorized based on their total scores as follows: very good studies (9–10 points), good studies (7–8 points), satisfactory studies (5–6 points), and unsatisfactory studies (0–4 points). The distribution of quality scores across all included studies was subsequently analyzed.

## Results

After applying the inclusion and exclusion criteria, we initially identified 1608 unique studies in PubMed, 1053 studies in Embase, and 682 studies in the Cochrane Library, and 59 additional studies through other sources. Review of titles, abstracts, and duplicate studies eliminated 2906 studies. The full text of the remaining 496 studies was reviewed, eliminating another 80 studies primarily due to an unmatched study population, a lack of data on respective phenotypes or respective testing tools, or the lack of pathogenicity descriptions for the identified variants. A total of 416 studies (NDD, 249; epilepsy, 167; Supplementary Table 2) with 124,937 participants were included in the random-effects meta-analysis. The screening and selection process is shown in Fig. 1.

The diagnostic yield of the main cohorts is presented in Table 1 and Supplementary Figure. CMA was implemented in 180 studies, TS in 116 studies, WES in 152 studies, reanalysis of sequencing data in 11 studies, and WGS in 24 studies. The pooled diagnostic yield of the NDDs cohort was 14.8% (95% confidence interval [CI], 13.4–16.2%; *I*^2^ = 94.5%) for CMA, 23.2% (95% CI, 20.2–26.6%; *I*^2^ = 86.8%) for TS, 35.3% (95% CI, 31.5–39.3%; *I*^2^ = 92.9%) for WES, 31.1% (95% CI, 28.3–34.2%; *I*^2^ = 93.2%) for the combination of TS and WES (TS/WES), and 30.5% (95% CI, 22.8–39.4%; *I*^2^ = 96.1%) for WGS. For the epilepsy cohort, the pooled diagnostic yield was 13.3% (95% CI, 11.2–15.7%; *I*^2^ = 79.5%) for CMA, 24.0% (95% CI, 21.5–26.7%; *I*^2^ = 92.3%) for TS, 34.2% (95% CI, 30.9–37.6%; *I*^2^ = 86.3%) for WES, 28.7% (95% CI, 26.4–31.0%; *I*^2^ = 92.7%) for TS/WES, and 39.1% (95% CI, 32.4–46.2%; *I*^2^ = 71.5%) for WGS. Reanalysis of the sequencing data provided an additional pooled diagnostic yield of 10.6% (95% CI, 7.2–15.4%; *I*^2^ = 76.9%) in the NDDs cohort and 10.4% (95% CI, 4.0–24.2%; *I*^2^ = 88.5%) in the epilepsy cohort.

**Fig. 2:**
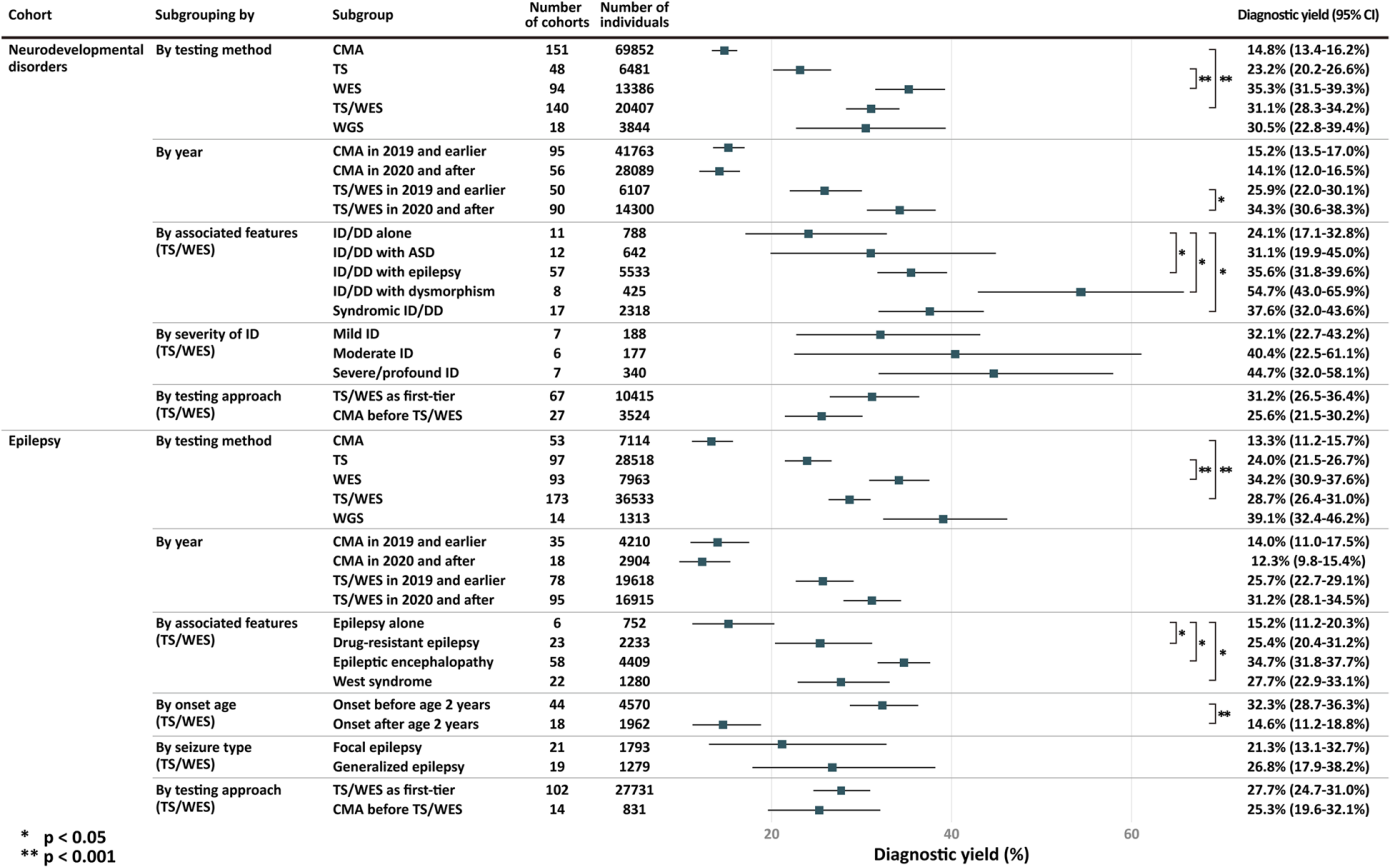
Diagnostic yield comparison between tests and cohorts. CMA, chromosomal microarray; TS, targeted sequencing; WES, whole-exome sequencing; WGS, whole-genome sequencing; ID, intellectual disability; DD, developmental delay

Comparison of the diagnostic yields between genetic tests (Fig. 2 and Table 1) showed that the pooled diagnostic yield of WES was significantly higher than that of TS in both the NDDs (35.3% vs. 23.2%; *p* < 0.0001) and epilepsy cohorts (34.2% vs. 24.0%; *p* < 0.0001). The pooled diagnostic yield of TS/WES was significantly higher than that of CMA in both the NDDs cohort (31.1% vs. 14.8%; *p* < 0.0001) and the epilepsy cohort (28.7% vs. 13.3%; *p* < 0.0001).

**Fig. 3:**
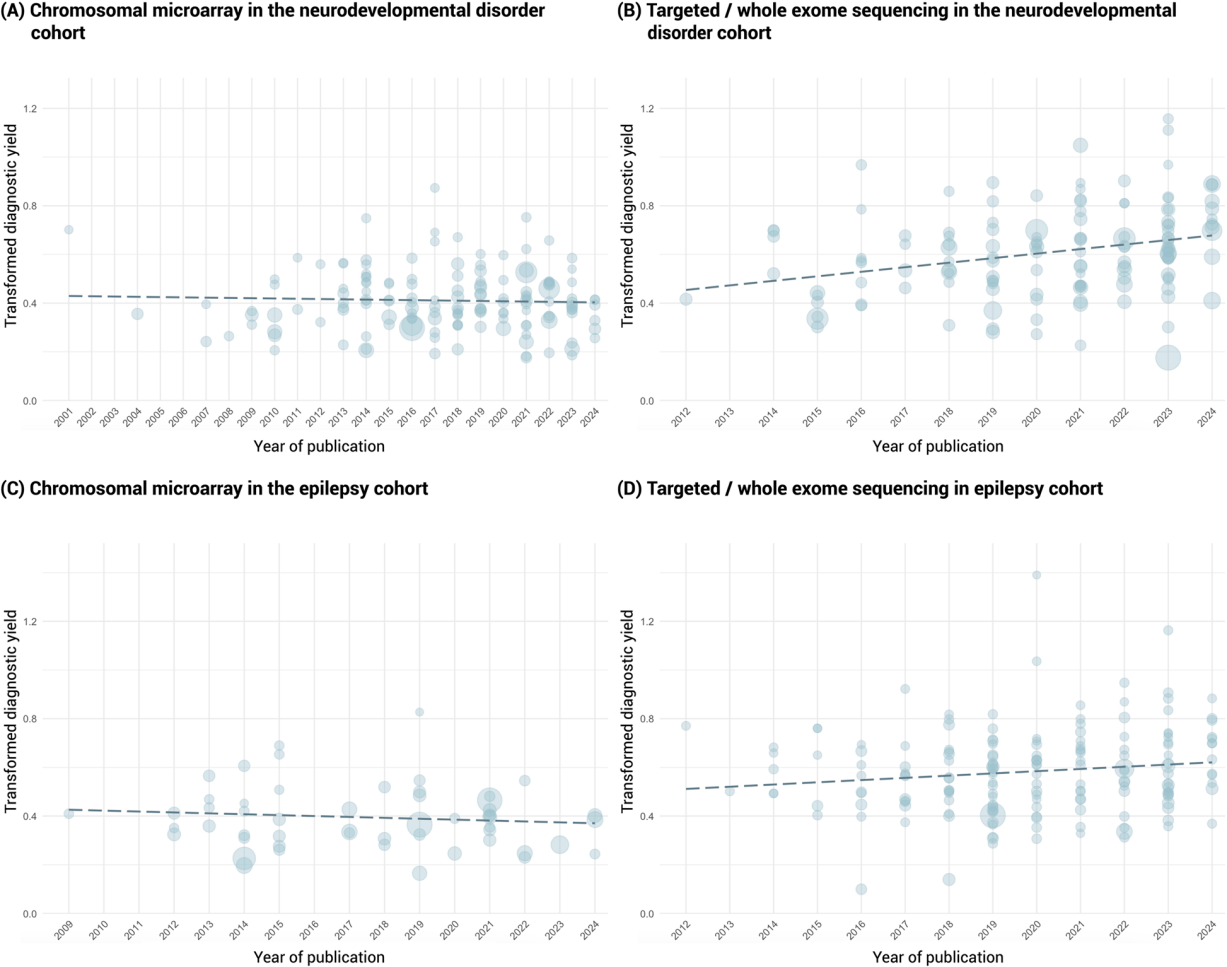
Diagnostic yield changes over the years according to Freeman-Tukey Double Arcsine Transformation. The figure illustrates the association between diagnostic yield and year of publication in chromosomal microarray and targeted/whole-exome sequencing in the two main cohorts. The size of the circles in the plot corresponds to the sample size for each study, and the dotted line is the regression line. The detailed study information is provided in the supplementary figure and supplementary table. For stabilizing the variance, the Freeman-Tukey double arcsine transformation is applied to the individual diagnostic yield with the transformed yield on the *y*-axis

Considering the change in diagnostic yield with time (Table 1; Figs. 2 and 3), we observed a similar yield for CMA between studies published in 2019 and earlier and in 2020 and after in both the NDDs and epilepsy cohorts. However, for TS/WES, we observed a significantly higher yield from the studies published in 2020 and after for both the NDDs cohort (34.3% vs. 25.9%; *p* < 0.01) and the epilepsy cohort (31.2% vs. 25.7%; *p* = 0.034).

The pooled diagnostic yields of CMA and TS/WES in each subgroup are presented in Table 2. The yields for TS/WES in mild, moderate, and severe/profound ID were 32.1% (95% CI, 22.7–43.2%), 40.4% (95% CI, 22.5–61.1%), and 44.7% (95% CI, 32.0–58.1%), respectively. In the analysis of ID/DD sorted by associated features, the pooled diagnostic yield of TS/WES was highest in ID/DD with dysmorphism, reaching 54.7% (95% CI, 43.0–65.9%; *p* = 0.003 when compared with isolated ID/DD); the yield was 37.6% (95% CI, 32.0–43.6%; *p* = 0.019) for ID/DD with syndromic presentation, 35.6% (95% CI, 31.8–39.6%; *p* = 0.033) for ID/DD with epilepsy, 31.1% (95% CI, 19.9–45.0%) for ID/DD with ASD, and 24.1% (95% CI, 17.1–32.8%) for isolated ID/DD. Among the epilepsy subgroups, the yields for TS/WES were 25.4% (95% CI, 20.4–31.2%, *p* = 0.043 when compared with isolated epilepsy) for drug-resistant epilepsy, 34.7% (95% CI, 31.8–37.7%;* p* = 0.0003) for epileptic encephalopathies, 27.7% (95% CI, 22.9–33.1%, *p* = 0.031) for West syndrome/infantile spasms, and 15.2% (95% CI, 11.2–20.3%) for isolated epilepsy. Considering the age at onset of epilepsy, the pooled diagnostic yield of TS/WES was 32.9% (95% CI, 29.1–36.9%) for individuals with onset earlier than 2 years of age, which was significantly higher than for those with onset after childhood (15.6%; 95% CI, 11.8–20.4%; *p* < 10^−4^). The yield of TS/WES was 21.3% (95% CI, 13.1–32.7%) for focal epilepsy and 26.8% (95% CI, 17.9–38.2%) for generalized epilepsy.

We compared the diagnostic yield for TS/WES between studies that used TS/WES as the first-tier test and those that applied TS/WES after a negative CMA result (Fig. 2). In the NDD cohort, the yield was 31.2% (95% CI, 26.5–36.4%) in the first-tier group and 25.6% (95% CI, 21.5–30.2%) in the group where CMA was used first (*p* = 0.12). In the epilepsy cohort, the yields were 27.7% (95% CI, 24.7–31.0%) and 25.3% (95% CI, 19.6–32.1%), respectively (*p* = 0.62).

A total of 381 out of 416 enrolled studies with full-text articles were evaluated using the NOS for cross-sectional studies, with detailed quality assessments presented in the Supplementary Table 3. The quality assessment revealed that 235 articles scored 7 points, 121 articles scored 8 points, 23 articles scored 9 points, and 2 articles scored 10 points. These results indicate that the majority of included studies were of good to very good methodological quality. Among the included studies, 132 articles met our criterion for adequate sample size (>200 participants). In terms of comparability assessment, 50 studies conducted further multivariate analysis or logistic regression analyses with clinical or genotype findings.

## Discussion

Here, we present a systematic review and meta-analysis of 416 studies comprising 124,937 individuals, of which 96,271 presented with NDDs and 49,765 with epilepsy. We analyzed the diagnostic yield of different genetic tests, including CMA and various sequencing technologies, and found that the yield of sequencing methods (TS/WES) was significantly higher than that of CMA in patients with NDDs or epilepsy. We also observed that the pooled diagnostic yield of WES was significantly higher than that of TS in NDDs and epilepsy. This result supports the previous recommendation to use exome sequencing as a first-tier test for patients with NDDs or epilepsy [7, 13].

While the sequencing method yielded more diagnostic results than did CMA in this study, sequencing theoretically cannot replace CMA since the two techniques are methodologically distinct in their variant detection capabilities. CMA is superior for detecting CNVs, whereas sequencing is more precise in the identification of SNVs and indels [14]. Reports have shown that relevant pathogenic CNVs were present in 10% of patients with NDDs and were critical in the pathogenesis of NDDs [15]. Therefore, while NGS provided a higher yield and could serve as a first-tier test for patients with NDDs, CMA or WGS should be considered for detecting causative CNVs if the TS/WES result is inconclusive.

We observed a significantly higher pooled diagnostic yield from exome sequencing (TS/WES) over time, as evidenced by the pooled diagnostic yield from studies published in 2019 and earlier compared to those in 2020 and after. Furthermore, the yield in the epilepsy cohort using TS/WES (28.7%) in our analysis was higher than that in another meta-analysis (24.0%) published in 2021 [8]. The increasing yield over time may be attributable to updates in various databases and literature, along with advances in bioinformatic analytical pipelines in recent years. Moreover, while accuracy still requires further validation, the development of algorithms for calling CNVs from sequencing data has challenged the traditional view that CNVs are undetectable by WES [16]. Additionally, while CMA can detect CNVs larger than 50 kb, CNV calling from sequencing data can identify smaller deletions/duplications that span single or multiple exons, thereby further increasing the diagnostic yield [14, 17, 18]. In this study, we noted a slightly higher yield of TS/WES in the NDD cohort when applying NGS as the first-tier test as compared to using CMA before NGS (31.2% vs. 25.6%). This result could be due to the ability of NGS to detect CNVs, albeit not completely. In the epilepsy cohort, the yields from the two sequential approaches were similar, suggesting that CNVs play a more significant role in the pathogenesis of NDD than in epilepsy.

In this study, we characterized the diagnostic yield of subgroups of NDDs and epilepsy. In considering the severity of NDDs, the yield of TS/WES is highest in patients with severe/profound ID, moderate in those with moderate ID, and lowest in those with mild ID, though not reaching statistical significance. This finding is consistent with previous study results [19]. Considering the associated features of ID/DD, a previous meta-analysis showed a higher, though not statistically significant, pooled diagnostic yield in the NDD plus associated conditions category as compared to those with NDD alone [7]. Our study further subcategorized the associated conditions, and the results suggest that epilepsy, facial dysmorphism, or syndromic presentations may significantly enrich the diagnostic yield. In the epilepsy cohort, the presence of epileptic encephalopathy, an earlier age of onset, and resistance to multiple antiseizure medications are features associated with a significantly higher yield of TS/WES, which is consistent with the previous meta-analysis [8]. These findings underscore the importance of detailed clinical phenotyping, which can guide more targeted test selection and data interpretation [20]. Detailed clinical phenotyping involves the systematic and thorough documentation of developmental milestones, neurologic and behavioral features, growth parameters, dysmorphic signs, radiologic findings, and coexisting medical conditions [21, 22]. By accurately characterizing these features, clinicians and geneticists can select the most appropriate genetic testing approach and optimize variant interpretation by focusing on genes, variant types, and pathways most relevant to the observed phenotype [23]. Moreover, in cases where VUS are identified, clearly delineated clinical presentations can help refine pathogenicity assessments and improve diagnostic confidence [24].

Reanalysis of previous exome sequencing data using the latest bioinformatic pipelines, allele frequency databases, and literature may provide an additional 5–26% in diagnostic yield [6]. Furthermore, the addition of validated gene-disease relationships in public databases—such as OMIM, ClinVar, or DECIPHER—likely contributes to this improvement in diagnostic yield [14]. Our study demonstrates an additional yield of around 10% in both the NDDs and epilepsy cohorts. Clinicians or geneticists may consider reanalyzing the existing sequencing data when there are newly developed phenotypes, additional information on family history, or newly discovered genes of interest, or when sufficient time has elapsed since the last analysis [9]. In addition, if the previous analysis did not incorporate CNV calling, reanalyzing the sequencing data using appropriate CNV callers could enrich diagnostic yields [16]. Moreover, reanalysis could be performed in an automated manner to periodically incorporate the latest databases and annotations, which further improves the cost efficiency [25]. Such periodic reanalysis is particularly relevant given the rapid expansion of genotype-phenotype correlation databases and literature, increasing the likelihood of identifying causative variants that were once classified as variants of unknown significance.

WGS is a powerful and comprehensive genetic test that can detect structural variants, CNVs, indels, and SNVs in both exon and intron regions [14]. Comparing WGS with WES/TS, our study demonstrated a similar pooled diagnostic yield in the NDDs cohort (31.1% vs. 30.5%; *p* = 0.76) and a significantly higher pooled diagnostic yield in the epilepsy cohort (39.1% vs. 28.7%; *p* = 0.027). The limited improvement in the yield of the NDDs cohort may be related to challenges in the interpretation and prioritization of non-coding variants because only a small portion of these variants had been confirmed to cause diseases, and most patients who receive a molecular diagnosis from WGS harbor variants in the coding region that theoretically could be detected by WES [26]. A recently published study using WGS for diagnosing rare diseases revealed that 72% of the causative variants were theoretically detectable with WES. On the other hand, WGS is required in detecting structural variants, specific coding regions, tandem repeat expansion, and deep intronic variants, constituting 8.2% of causative variants [20]. However, the number of studies on WGS currently is still scarce, and more studies are warranted to determine the actual diagnostic yield of WGS in individuals with NDD or epilepsy. As larger sample sizes and improved analytical pipelines become available, the full advantage of WGS—including its potential to elucidate noncoding region pathogenic variants—may become more evident, especially for complex phenotypes with previously undiagnosed etiologies [20].

In comparison with previous meta-analyses [7, 8, 13], our study has several important new contributions. First, we have included the largest cohort to date, with over 124,000 total participants. Second, we have explicitly incorporated WGS data and reanalysis cohorts, which have been less emphasized in earlier meta-analyses, to provide a more comprehensive evaluation of diagnostic yield. Notably, our results show that WGS offers a higher diagnostic yield in epilepsy than WES/TS, a finding that was either not reported or insufficiently powered in prior studies. In addition, our estimate of approximately 10% incremental yield from reanalysis further underscores the value of periodic reanalysis. Although our findings, consistent with earlier studies, suggest that WES/TS provides a higher diagnostic yield compared to CMA, our significantly larger sample size leads to increased statistical power and robustness to this conclusion. Consequently, these results offer a more reliable evidence base to inform clinical practice and guidelines.

In line with the previous meta-analysis, our study showed high *I*^2^ values, ranging between 71.5% and 96.1%, which suggests high heterogeneity [8, 27]. This heterogeneity may arise from study differences in the reporting center, time, bioinformatic pipeline (e.g., CNV calling), genes incorporated in the targeted sequencing, or the included participant criteria (e.g., consanguinity). In addition, the yield of proband-only or trio-based sequencing may differ. Moreover, the yield of a genetic test that was performed after another unrevealing test (e.g., WES after a negative TS) may be lower than that of directly performing the test. Of note, the high *I*^2^ values could also be a reflection of the study design and the study population, as stated in a study showing that the nature of proportional meta-analysis, the noncomparative design of each study, and the large sample size may all contribute to a high *I*^2^ value [28].

In this systematic review, we rigorously assessed the methodological quality of 381 included studies with full text using the Newcastle-Ottawa Scale adapted for cross-sectional studies. A notable strength of our review is the comprehensive quality assessment of studies reporting diagnostic yield rates, an aspect often overlooked in previous reviews. The majority of included studies demonstrated good to very good methodological quality. However, several limitations should be acknowledged. Despite the generally high methodological scores, we found that approximately 65.4% (249/381) of the included studies had sample sizes below our threshold of 200 participants, potentially limiting the generalizability of their findings. Furthermore, only 50 studies conducted further multivariate analysis or logistic regression analyses with clinical or genotype findings, with most studies employing basic statistical comparisons. This predominance of simpler statistical approaches might have restricted the depth of analysis regarding potential confounding factors and effect modifiers in the diagnostic yield rates.

Our study is also limited by several factors. First, not all studies follow the ACMG guidelines for the interpretation of a variant, and the standard of causative variant determination might differ between researchers. Individuals who undergo genetic tests may be preselected by clinicians based on different criteria, clinical scenarios, or personal experiences; thus, the yield may deviate from the actual yield of individuals with NDDs or epilepsy. Moreover, the definition of each associated condition (such as dysmorphism, syndromic presentation, or epileptic encephalopathy) may differ between the studies, thereby causing heterogeneity between study cohorts. Additionally, a low number of studies were identified in certain cohorts, especially those involving WGS. Future studies using standardized patient selection criteria, analytic pipelines and databases, and testing methodology using a large sample size are warranted to minimize heterogeneity.

NDDs and epilepsy encompass a broad genetic landscape, ranging from monogenic causes to polygenic or multifactorial etiologies [4]. Our results primarily focus on detecting monogenic causes, thus highlighting the diagnostic yield of sequencing-based methods in this subset of patients. However, individuals who remain undiagnosed despite thorough monogenic testing may harbor complex diseases attributed to polygenic risk factors or common genomic variants. As genome-wide association studies (GWAS) continue to expand in sample size, the genomic regions contributing to these complex, polygenic disorders will become more clearly defined, reducing the need for sequencing-based technologies to detect such common risk variants [29]. In this context, array-based methods and the application of polygenic risk scores can be particularly beneficial for elucidating risk profiles and guiding management in patients whose phenotypes are driven by the accumulation of multiple, smaller-effect alleles rather than a single pathogenic variant [30]. Thus, while exome or genome sequencing remains an essential tool for rare monogenic cases, a balanced approach that integrates genotyping-based data and emerging polygenic assessment strategies is crucial for addressing the full spectrum of neurodevelopmental and epilepsy disorders.

In conclusion, we conducted a meta-analysis comparing the diagnostic yield of CMA and various sequencing technologies between individuals with NDDs or epilepsy. Sequencing methods provided a significantly higher pooled diagnostic yield than did CMA, supporting the recommendations that exome or genome sequencing be the first-tier test for individuals with NDDs or epilepsy. Patients with NDDs associated with dysmorphism or syndromic presentation, and patients with epilepsy associated with onset earlier than 2 years of age, drug-resistant epilepsy, and epileptic encephalopathy may particularly benefit from exome sequencing due to its higher yield.

## Supplementary Information

Supplementary Material 1.

## Data Availability

All data generated or analyzed during this study are included in this published article and the supplementary files.

## Abbreviations

NDDs: Neurodevelopmental disorders
ID: Intellectual disability
DD: Developmental delay
ASD: Autism spectrum disorder
CNVs: Copy number variants
indels: Small insertions or deletions
CMA: Chromosomal microarray
NGS: Next-generation sequencing
TS: Targeted sequencing
WES: Whole exome sequencing
WGS: Whole genome sequencing
VUS: Variant of unknown significance
ACMG: American College of Medical Genetics and Genomics
PRISMA: Preferred Reporting Items for Systematic Reviews and Meta-Analyses
CI: Confidence interval
