# Supplementary material for: Genetic testing for diagnosing neurodevelopmental disorders and epilepsy: a systematic review and meta-analysis
Source: Syst Rev. 2025 Jul 28;14:155. doi: 10.1186/s13643-025-02896-y (PMC12302900; doi:10.1186/s13643-025-02896-y)
Supplement: Supplementary file 1 — Supplementary Material 1. [file 13643_2025_2896_MOESM1_ESM.pdf]

Supplemental Materials

Supplementary Figure. Forest plots of the main cohort

Supplementary Table 1. Search terms

Supplementary Table 2. The 416 articles included in this study

Supplementary Table 3. Newcastle-Ottawa Scale (NOS) Quality Assessment

Supplementary Figure. Forest plots of the main cohort. (A, B) Individuals with neurodevelopmental disorders undergoing (A) chromosomal microarray (CMA) or (B) targeted sequencing or whole exome sequencing (TS/WES). (C, D) Individuals with epilepsy undergoing (C) CMA or (D) TS/WES.

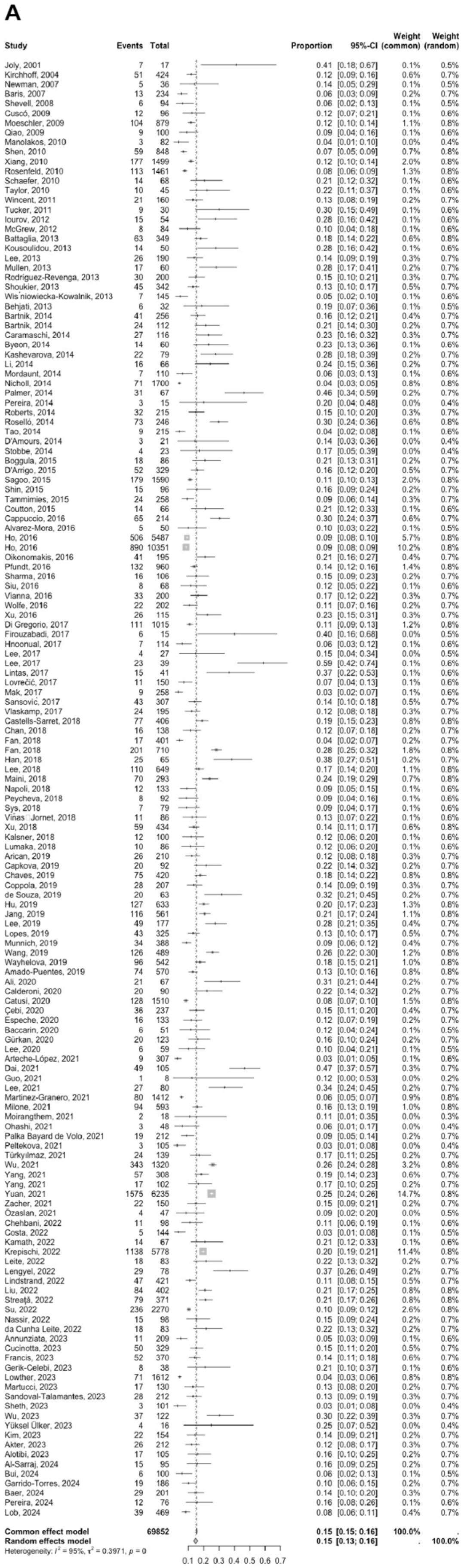

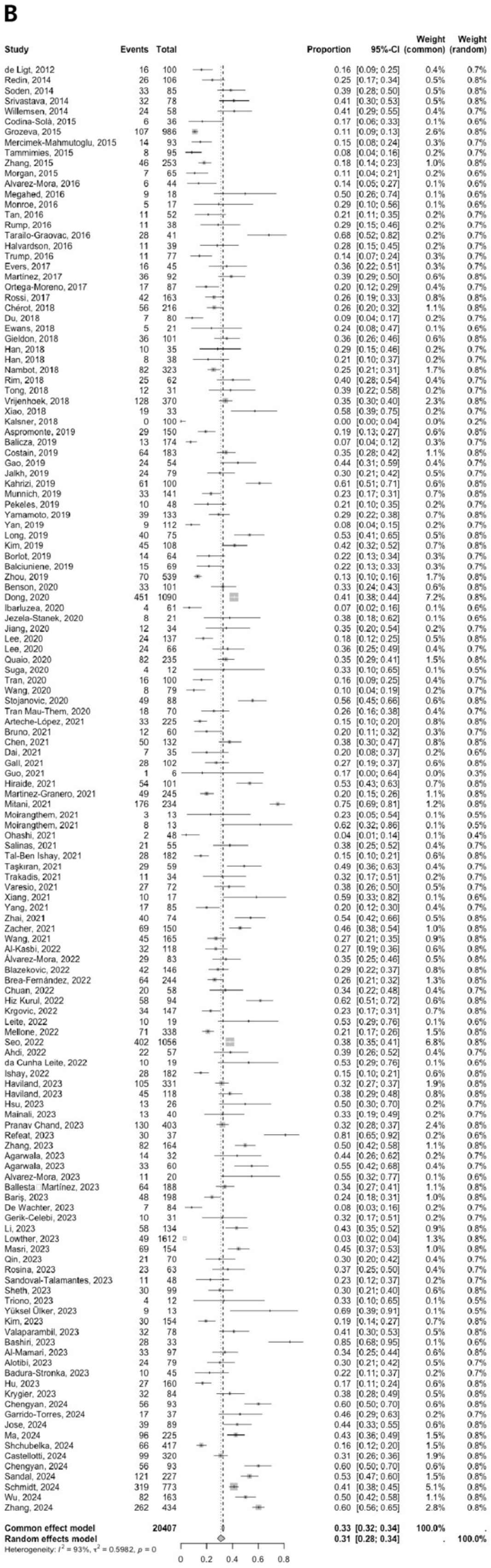

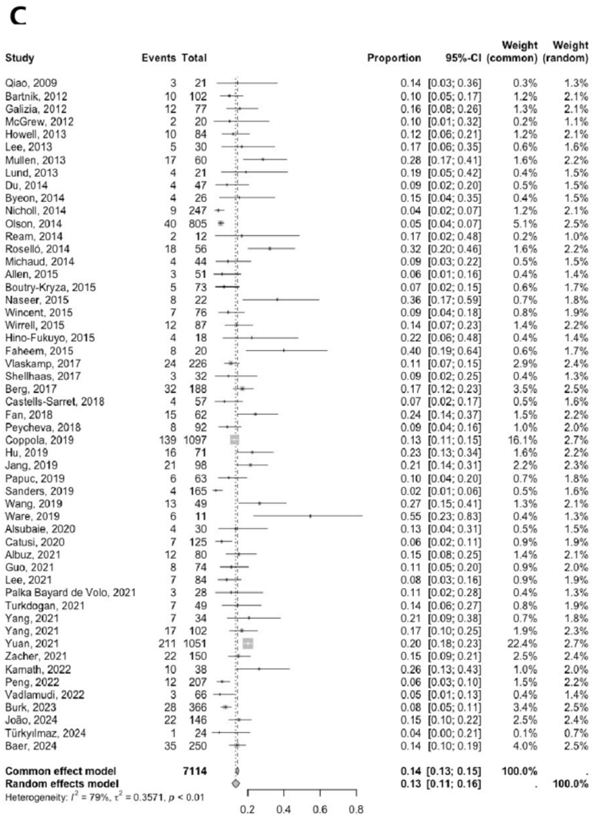
C
Study
Events
Total
Proportion
95%-CI
Weight (common)
Weight (random)
Common effect model
Random effects model

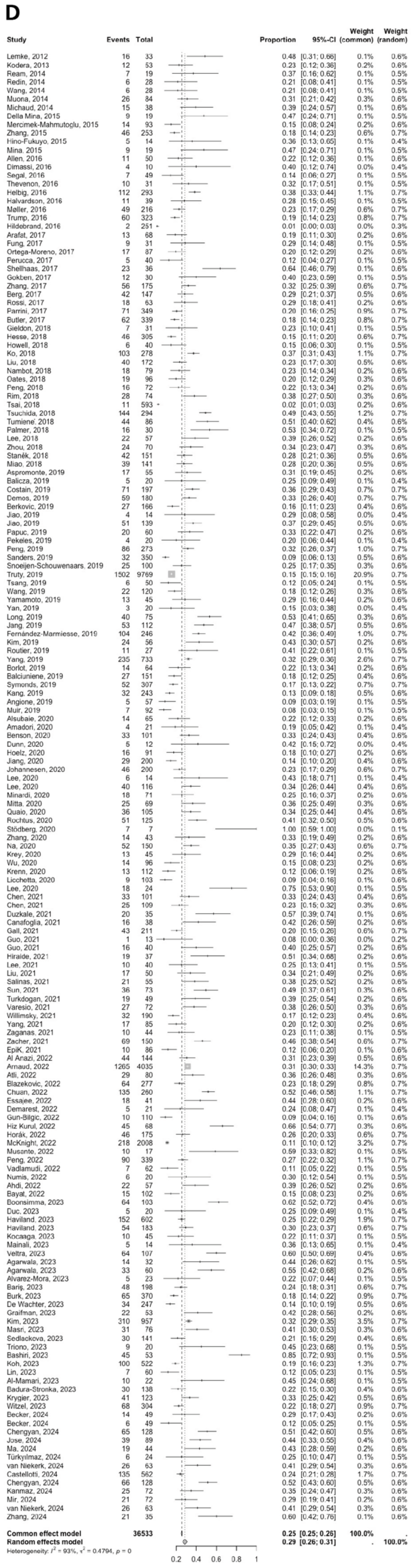
D
Study
Events
Total
Proportion
95%-CI
Weight (common)
Weight (random)
Common effect model
Random effects model

Supplementary Table 1. Search terms

	Terms
#1	(epilepsy) OR (epileptic encephalopathy) OR (seizures)
#2	(neurodevelopmental disorder) OR (intellectual disability) OR (ID) OR (mental retardation) OR (developmental delay)
#3	(autism) OR (ASD) OR (autism spectrum disorder)
#4	((chromosom* OR oligonucleotide OR "deoxyribonucleic acid" OR DNA OR gene OR SNP OR "single nucleotide polymorphism") adj2 (array OR microarray OR microchip* OR chip*)) OR CMA OR aCGH OR CGH OR "comparative genome hybridization"
#5	(exome adj3 sequenc*) OR WES OR next-generation OR "next generation" OR NGS
#6	(genome adj3 sequenc*) OR WGS
#7	(cohort) OR (diagnostic yield) OR (diagnostic test) OR (clinical practice)
Combined Query	((#1 OR #2 OR #3) AND (#4 OR #5 OR #6)) AND #7

Supplementary Table 2. The 416 articles included in this study

1	The Epilepsy Genetics Initiative: Systematic reanalysis of diagnostic exomes increases yield. Epilepsia. 2019;60(5):797-806.
2	Abdi M, Aliyev E, Trost B, Kohailan M, Aamer W, Syed N, et al. Genomic architecture of autism spectrum disorder in Qatar: The BARAKA-Qatar Study. Genome Med. 2023;15(1):81.
3	Abe-Hatano C, Iida A, Kosugi S, Momozawa Y, Terao C, Ishikawa K, et al. Whole genome sequencing of 45 Japanese patients with intellectual disability. American Journal of Medical Genetics, Part A. 2021;185(5):1468-80.
4	Agarwala P, Narang B, Geetha TS, Kurwale N, Samson PL, Golani T, et al. Early-infantile developmental and epileptic encephalopathy: the aetiologies, phenotypic differences and outcomes-a prospective observational study. Brain Commun. 2023;5(5):fcad243.
5	Ahdi SG, Sultan T. Genotype-Phenotype Correlations in Epileptic Encephalopathy Syndromes in Less Than 2 Years of Age: A Single Centre Experience. Pakistan Paediatric Journal. 2022;46(4):417-23.
6	Akter H, Rahman MM, Sarker S, Basiruzzaman M, Islam MM, Rahaman MA, et al. Construction of copy number variation landscape and characterization of associated genes in a Bangladeshi cohort of neurodevelopmental disorders. Frontiers in Genetics. 2023;14.
7	Al Anazi AH, Ammar AS, Al-Hajj M, Cyrus C, Aljaafari D, Khoda I, et al. Whole-exome sequencing of a Saudi epilepsy cohort reveals association signals in known and potentially novel loci. Hum Genomics. 2022;16(1):71.
8	Al-Kasbi G, Al-Murshedi F, Al-Kindi A, Al-Hashimi N, Al-Thihli K, Al-Saegh A, et al. The diagnostic yield, candidate genes, and pitfalls for a genetic study of intellectual disability in 118 middle eastern families. Sci Rep. 2022;12(1):18862.
9	Al-Mamari W, Idris AB, Al-Thihli K, Abdulrahim R, Jalees S, Al-Jabri M, et al. Applying whole exome sequencing in a consanguineous population with autism spectrum disorder. International Journal of Developmental Disabilities. 2023;69(2):190-200.
10	Al-Nabhani M, Al-Rashdi S, Al-Murshedi F, Al-Kindi A, Al-Thihli K, Al-Saegh A, et al. Reanalysis of exome sequencing data of intellectual disability samples: Yields and benefits. Clin Genet. 2018;94(6):495-501.
11	Al-Sarraj Y, Taha RZ, Al-Dous E, Ahram D, Abbasi S, Abuazab E, et al. The genetic landscape of autism spectrum disorder in the Middle Eastern population. Front Genet. 2024;15:1363849.
12	Albuz B, Ozdemir O, Silan F. The high frequency of chromosomal copy number variations and candidate genes in epilepsy patients. Clin Neurol Neurosurg. 2021;202:106487.
13	Ali MAM, Hassan AM, Saafan MA, Abdelmagid AA. Additive Diagnostic Yield of Homozygosity Regions Identified During Chromosomal microarray Testing in Children with Developmental Delay, Dysmorphic Features or Congenital Anomalies. Biochem Genet. 2020;58(1):74-101.
14	Allen AS, Aggarwal V, Berkovic SF, Cossette P, Delanty N, Dlugos D, et al. Diverse genetic causes of polymicrogyria with epilepsy. Epilepsia. 2021;62(4):973-83.
15	Allen NM, Conroy J, Shahwan A, Ennis S, Lynch B, Lynch SA, et al. Chromosomal microarray in unexplained severe early onset epilepsy - A single centre cohort. Eur J Paediatr Neurol. 2015;19(4):390-4.
16	Allen NM, Conroy J, Shahwan A, Lynch B, Correa RG, Pena SD, et al. Unexplained early onset

	epileptic encephalopathy: Exome screening and phenotype expansion. Epilepsia. 2016;57(1):e12-7.
17	Alotibi RS, Sannan NS, AlEissa M, Aldriwesh MG, Al Tuwaijri A, Akiel MA, et al. The diagnostic yield of CGH and WES in neurodevelopmental disorders. Front Pediatr. 2023;11:1133789.
18	Alsubaie L, Aloraini T, Amoudi M, Swaid A, Eyiad W, Al Mutairi F, et al. Genomic testing and counseling: The contribution of next-generation sequencing to epilepsy genetics. Ann Hum Genet. 2020;84(6):431-6.
19	Alvarez-Mora MI, Calvo Escalona R, Puig Navarro O, Madrigal I, Quintela I, Amigo J, et al. Comprehensive molecular testing in patients with high functioning autism spectrum disorder. Mutat Res. 2016;784-785:46-52.
20	Alvarez-Mora MI, Rodríguez-Revenga L, Jodar M, Potrony M, Sanchez A, Badenas C, et al. Implementation of Exome Sequencing in Clinical Practice for Neurological Disorders. Genes (Basel). 2023;14(4).
21	Álvarez-Mora MI, Sánchez A, Rodríguez-Revenga L, Corominas J, Rabionet R, Puig S, et al. Diagnostic yield of next-generation sequencing in 87 families with neurodevelopmental disorders. Orphanet J Rare Dis. 2022;17(1):60.
22	Amado-Puentes A, Reparaz-Andrade A, Del Campo-García A, Blanco-Barca MÓ, Salgado-Barreira Á, Del Campo-Pérez V, et al. Neurodevelopmental Disorders and Array-Based Comparative Genomic Hybridization: Sensitivity and Specificity using a Criteria Checklist for Genetic Test Performance. Neuropediatrics. 2019;50(3):164-9.
23	Amadori E, Scala M, Cereda GS, Vari MS, Marchese F, Di Pisa V, et al. Targeted re-sequencing for early diagnosis of genetic causes of childhood epilepsy: the Italian experience from the 'beyond epilepsy' project. Ital J Pediatr. 2020;46(1):92.
24	Angione K, Eschbach K, Smith G, Joshi C, Demarest S. Genetic testing in a cohort of patients with potential epilepsy with myoclonic-atonic seizures. Epilepsy Res. 2019;150:70-7.
25	Annunziata S, Bulgheroni S, D'Arrigo S, Esposito S, Taddei M, Saletti V, et al. CGH Findings in Children with Complex and Essential Autistic Spectrum Disorder. J Autism Dev Disord. 2023;53(2):615-23.
26	Arafat A, Jing P, Ma Y, Pu M, Nan G, Fang H, et al. Unexplained Early Infantile Epileptic Encephalopathy in Han Chinese Children: Next-Generation Sequencing and Phenotype Enriching. Sci Rep. 2017;7:46227.
27	Arican P, Olgac Dundar N, Ozyilmaz B, Cavusoglu D, Gencpinar P, Erdogan KM, et al. Chromosomal Microarray Analysis in Children with Unexplained Developmental Delay/Intellectual Disability. J Pediatr Genet. 2019;8(1):1-9.
28	Arnaud L, Abi Warde MT, Barcia G, de Bellescize J, Chatron N, Faoucher M, et al. The EPIGENE network: A French initiative to harmonize and improve the nationwide diagnosis of monogenic epilepsies. Eur J Med Genet. 2022;65(3):104445.
29	Arteche-López A, Gómez Rodríguez MJ, Sánchez Calvin MT, Quesada-Espinosa JF, Lezana Rosales JM, Palma Milla C, et al. Towards a Change in the Diagnostic Algorithm of Autism Spectrum Disorders: Evidence Supporting Whole Exome Sequencing as a First-Tier Test. Genes (Basel). 2021;12(4).
30	Aspromonte MC, Bellini M, Gasparini A, Carraro M, Bettella E, Polli R, et al. Characterization of

	intellectual disability and autism comorbidity through gene panel sequencing. Hum Mutat. 2019;40(9):1346-63.
31	Atli EI, Atli E, Yalcintepe S, Demir S, Kalkan R, Eker D, et al. Customised targeted massively parallel sequencing enables more precise diagnosis of patients with epilepsy. Intern Med J. 2022;52(7):1174-84.
32	Baccarin M, Picinelli C, Tomaiuolo P, Castronovo P, Costa A, Verdecchia M, et al. Appropriateness of array-CGH in the ADHD clinics: A comparative study. Genes Brain Behav. 2020;19(6):e12651.
33	Badura-Stronka M, Wołyńska K, Winczewska-Wiktor A, Marcinkowska J, Karolewska D, Tomkowiak-Kędzia D, et al. Validation of targeted next-generation sequencing panels in a cohort of Polish patients with epilepsy: assessing variable performance across clinical endophenotypes and uncovering novel genetic variants. Frontiers in Neurology. 2023;14.
34	Baer S, Schalk A, Miguet M, Schaefer É, El Chehadeh S, Ginglinger E, et al. Copy Number Variation and Epilepsy: State of the Art in the Era of High-Throughput Sequencing-A Multicenter Cohort Study. Pediatr Neurol. 2024;159:16-25.
35	Bakshi M. Application of Whole Genome Sequencing for diagnosis of Intellectual disability in a multiethnic cohort-Initial findings and reanalysis. Unsworks. 2020.
36	Balciuniene J, DeChene ET, Akgumus G, Romasko EJ, Cao K, Dubbs HA, et al. Use of a Dynamic Genetic Testing Approach for Childhood-Onset Epilepsy. JAMA Netw Open. 2019;2(4):e192129.
37	Balicza P, Varga N, Bolgár B, Pentelényi K, Bencsik R, Gál A, et al. Comprehensive Analysis of Rare Variants of 101 Autism-Linked Genes in a Hungarian Cohort of Autism Spectrum Disorder Patients. Front Genet. 2019;10:434.
38	Ballesta-Martínez MJ, Pérez-Fernández V, López-González V, Sánchez-Soler MJ, Serrano-Antón AT, Rodríguez-Peña LI, et al. Validation of clinical exome sequencing in the diagnostic procedure of patients with intellectual disability in clinical practice. Orphanet J Rare Dis. 2023;18(1):201.
39	Baris HN, Tan WH, Kimonis VE, Irons MB. Diagnostic utility of array-based comparative genomic hybridization in a clinical setting. Am J Med Genet A. 2007;143a(21):2523-33.
40	Bariş S, Kırık S, Balasar Ö. Importance of targeted next-generation sequencing in pediatric patients with developmental epileptic encephalopathy. Rev Assoc Med Bras (1992). 2023;69(10):e20230547.
41	Bartnik M, Nowakowska B, Derwińska K, Wiśniowiecka-Kowalnik B, Kędzior M, Bernaciak J, et al. Application of array comparative genomic hybridization in 256 patients with developmental delay or intellectual disability. J Appl Genet. 2014;55(1):125-44.
42	Bartnik M, Szczepanik E, Derwińska K, Wiśniowiecka-Kowalnik B, Gambin T, Sykulski M, et al. Application of array comparative genomic hybridization in 102 patients with epilepsy and additional neurodevelopmental disorders. Am J Med Genet B Neuropsychiatr Genet. 2012;159b(7):760-71.
43	Bartnik M, Wiśniowiecka-Kowalnik B, Nowakowska B, Smyk M, Kędzior M, Sobecka K, et al. The usefulness of array comparative genomic hybridization in clinical diagnostics of intellectual disability in children. Dev Period Med. 2014;18(3):307-17.
44	Bashiri FA, AlSheikh R, Hamad MH, Alsheikh H, Alsheikh RA, Kentab A, et al. Genotype-Phenotype Analysis of Children with Epilepsy Referred for Whole-Exome Sequencing at a Tertiary Care University Hospital. Children (Basel). 2023;10(8).
45	Battaglia A, Doccini V, Bernardini L, Novelli A, Loddo S, Capalbo A, et al. Confirmation of

	chromosomal microarray as a first-tier clinical diagnostic test for individuals with developmental delay, intellectual disability, autism spectrum disorders and dysmorphic features. Eur J Paediatr Neurol. 2013;17(6):589-99.
46	Bayanova M, Bolatov AK, Bazenova A, Nazarova L, Nauryzbayeva A, Tanko NM, et al. Whole-Genome Sequencing Among Kazakhstani Children with Early-Onset Epilepsy Revealed New Gene Variants and Phenotypic Variability. Mol Neurobiol. 2023;60(8):4324-35.
47	Bayat A, Fenger CD, Techlo TR, Højte AF, Nørgaard I, Hansen TF, et al. Impact of Genetic Testing on Therapeutic Decision-Making in Childhood-Onset Epilepsies—a Study in a Tertiary Epilepsy Center. Neurotherapeutics. 2022;19(4):1353-67.
48	Becker LL, Makridis KL, Abad-Perez AT, Thomale UW, Tietze A, Elger CE, et al. The importance of routine genetic testing in pediatric epilepsy surgery. Epilepsia Open. 2024;9(2):800-7.
49	Behjati F, Firouzabadi SG, Kariminejad R, Vameghi R, Sajedi F, Shafaghati Y, et al. Genomic characterization of some Iranian children with idiopathic mental retardation using array comparative genomic hybridization. Indian Journal of Human Genetics. 2013;19(4):443-8.
50	Benson KA, White M, Allen NM, Byrne S, Carton R, Comerford E, et al. A comparison of genomic diagnostics in adults and children with epilepsy and comorbid intellectual disability. Eur J Hum Genet. 2020;28(8):1066-77.
51	Berg AT, Coryell J, Saneto RP, Grinspan ZM, Alexander JJ, Kekis M, et al. Early-Life Epilepsies and the Emerging Role of Genetic Testing. JAMA Pediatr. 2017;171(9):863-71.
52	Blazekovic A, Gotovac Jercic K, Meglaj S, Duranovic V, Prpic I, Lozic B, et al. Genetics of Pediatric Epilepsy: Next-Generation Sequencing in Clinical Practice. Genes (Basel). 2022;13(8).
53	Boggula VR, Agarwal M, Kumar R, Awasthi S, Phadke SR. Recurrent benign copy number variants & issues in interpretation of variants of unknown significance identified by cytogenetic microarray in Indian patients with intellectual disability. Indian J Med Res. 2015;142(6):699-712.
54	Boonsimma P, Ittiwut C, Kamolvisit W, Ittiwut R, Chetruengchai W, Phokaew C, et al. Exome sequencing as first-tier genetic testing in infantile-onset pharmacoresistant epilepsy: diagnostic yield and treatment impact. Eur J Hum Genet. 2023;31(2):179-87.
55	Borlot F, de Almeida BI, Combe SL, Andrade DM, Filloux FM, Myers KA. Clinical utility of multigene panel testing in adults with epilepsy and intellectual disability. Epilepsia. 2019;60(8):1661-9.
56	Boutry-Kryza N, Labalme A, Ville D, de Bellescize J, Touraine R, Prieur F, et al. Molecular characterization of a cohort of 73 patients with infantile spasms syndrome. Eur J Med Genet. 2015;58(2):51-8.
57	Brea-Fernández AJ, Álvarez-Barona M, Amigo J, Tubío-Fungueiriño M, Caamaño P, Fernández-Prieto M, et al. Trio-based exome sequencing reveals a high rate of the de novo variants in intellectual disability. Eur J Hum Genet. 2022;30(8):938-45.
58	Bruno LP, Doddato G, Valentino F, Baldassarri M, Tita R, Fallerini C, et al. New Candidates for Autism/Intellectual Disability Identified by Whole-Exome Sequencing. Int J Mol Sci. 2021;22(24).
59	Bui HTP, Huy Do D, Ly HTT, Tran KT, Le HTT, Nguyen KT, et al. De novo copy number variations in candidate genomic regions in patients of severe autism spectrum disorder in Vietnam. PLoS One. 2024;19(3):e0290936.
60	Burk KC, Kaneko M, Quindipan C, Vu MH, Cepin MF, Santoro JD, et al. Diagnostic Yield of Epilepsy-Genes Sequencing and Chromosomal Microarray in Pediatric Epilepsy. Pediatr Neurol.

	2023;150:50-6.
61	Butler KM, da Silva C, Alexander JJ, Hegde M, Escayg A. Diagnostic Yield From 339 Epilepsy Patients Screened on a Clinical Gene Panel. Pediatr Neurol. 2017;77:61-6.
62	Byeon JH, Shin E, Kim GH, Lee K, Hong YS, Lee JW, et al. Application of array-based comparative genomic hybridization to pediatric neurologic diseases. Yonsei Med J. 2014;55(1):30-6.
63	Calderoni S, Ricca I, Balboni G, Cagiano R, Cassandrini D, Doccini S, et al. Evaluation of Chromosome Microarray Analysis in a Large Cohort of Females with Autism Spectrum Disorders: A Single Center Italian Study. J Pers Med. 2020;10(4).
64	Canafoglia L, Franceschetti S, Gambardella A, Striano P, Giallonardo AT, Tinuper P, et al. Progressive Myoclonus Epilepsies: Diagnostic Yield With Next-Generation Sequencing in Previously Unsolved Cases. Neurol Genet. 2021;7(6):e641.
65	Capkova P, Srovnal J, Capkova Z, Staffova K, Becvarova V, Trkova M, et al. MLPA is a practical and complementary alternative to CMA for diagnostic testing in patients with autism spectrum disorders and identifying new candidate CNVs associated with autism. PeerJ. 2019;6:e6183.
66	Cappuccio G, Vitiello F, Casertano A, Fontana P, Genesio R, Bruzzese D, et al. New insights in the interpretation of array-CGH: autism spectrum disorder and positive family history for intellectual disability predict the detection of pathogenic variants. Ital J Pediatr. 2016;42:39.
67	Caramaschi E, Stanghellini I, Magini P, Giuffrida MG, Scullin S, Giuva T, et al. Predictive diagnostic value for the clinical features accompanying intellectual disability in children with pathogenic copy number variations: a multivariate analysis. Ital J Pediatr. 2014;40:39.
68	Castellotti B, Ragona F, Freri E, Messina G, Magri S, Previtali R, et al. Next-generation sequencing in pediatric-onset epilepsies: Analysis with target panels and personalized therapeutic approach. Epilepsia Open. 2024.
69	Castells-Sarret N, Cueto-González AM, Borregan M, López-Grondona F, Miró R, Tizzano E, et al. [Comparative genomic hybridisation as a first option in genetic diagnosis: 1,000 cases and a cost-benefit analysis]. An Pediatr (Engl Ed). 2018;89(1):3-11.
70	Catusi I, Recalcati MP, Bestetti I, Garzo M, Valtorta C, Alfonsi M, et al. Testing single/combined clinical categories on 5110 Italian patients with developmental phenotypes to improve array-based detection rate. Mol Genet Genomic Med. 2020;8(1):e1056.
71	Çebi AH, Altıner Ş. Application of Chromosome Microarray Analysis in the Investigation of Developmental Disabilities and Congenital Anomalies: Single Center Experience and Review of NRXN3 and NEDD4L Deletions. Mol Syndromol. 2020;11(4):197-206.
72	Chan PY, Luk HM, Lee FM, Lo IF. Genetic profile and clinical application of chromosomal microarray in children with intellectual disability in Hong Kong. Hong Kong Med J. 2018;24(5):451-9.
73	Chaves TF, Baretto N, Oliveira LF, Ocampos M, Barbato IT, Anselmi M, et al. Copy Number Variations in a Cohort of 420 Individuals with Neurodevelopmental Disorders From the South of Brazil. Sci Rep. 2019;9(1):17776.
74	Chehbani F, Tomaiuolo P, Picinelli C, Baccarin M, Castronovo P, Scattoni ML, et al. Yield of array-CGH analysis in Tunisian children with autism spectrum disorder. Mol Genet Genomic Med. 2022;10(8):e1939.
75	Chen W, Qin J, Shen Y, Liang J, Cui Y, Zhang Y. Next generation sequencing in children with

	unexplained epilepsy: A retrospective cohort study. Brain Dev. 2021;43(10):1004-12.
76	Chengyan L, Chupeng X, You W, Yinhui C, Binglong H, Dang A, et al. Identification of genetic causes in children with unexplained epilepsy based on trio-whole exome sequencing. Clin Genet. 2024.
77	Chérot E, Keren B, Dubourg C, Carré W, Fradin M, Lavillaureix A, et al. Using medical exome sequencing to identify the causes of neurodevelopmental disorders: Experience of 2 clinical units and 216 patients. Clin Genet. 2018;93(3):567-76.
78	Chuan Z, Ruikun C, Qian L, Shiyue M, Shengju H, Yong Y, et al. Genetic and Phenotype Analysis of a Chinese Cohort of Infants and Children With Epilepsy. Front Genet. 2022;13:869210.
79	Codina-Solà M, Rodríguez-Santiago B, Homs A, Santoyo J, Rigau M, Aznar-Laín G, et al. Integrated analysis of whole-exome sequencing and transcriptome profiling in males with autism spectrum disorders. Mol Autism. 2015;6:21.
80	Coppola A, Cellini E, Stamberger H, Saarentaus E, Cetica V, Lal D, et al. Diagnostic implications of genetic copy number variation in epilepsy plus. Epilepsia. 2019;60(4):689-706.
81	Costa CIS, da Silva Montenegro EM, Zarrei M, de Sá Moreira E, Silva IMW, de Oliveira Scliar M, et al. Copy number variations in a Brazilian cohort with autism spectrum disorders highlight the contribution of cell adhesion genes. Clin Genet. 2022;101(1):134-41.
82	Costain G, Cordeiro D, Matviychuk D, Mercimek-Andrews S. Clinical Application of Targeted Next-Generation Sequencing Panels and Whole Exome Sequencing in Childhood Epilepsy. Neuroscience. 2019;418:291-310.
83	Coutton C, Dieterich K, Satre V, Vieville G, Amblard F, David M, et al. Array-CGH in children with mild intellectual disability: A population-based study. European Journal of Pediatrics. 2015;174(1):75-83.
84	Cucinotta F, Lintas C, Tomaiuolo P, Baccarin M, Picinelli C, Castronovo P, et al. Diagnostic yield and clinical impact of chromosomal microarray analysis in autism spectrum disorder. Mol Genet Genomic Med. 2023;11(8):e2182.
85	Cuscó I, Medrano A, Gener B, Vilardell M, Gallastegui F, Villa O, et al. Autism-specific copy number variants further implicate the phosphatidylinositol signaling pathway and the glutamatergic synapse in the etiology of the disorder. Hum Mol Genet. 2009;18(10):1795-804.
86	D'Amours G, Langlois M, Mathonnet G, Fetni R, Nizard S, Srour M, et al. SNP arrays: comparing diagnostic yields for four platforms in children with developmental delay. BMC Med Genomics. 2014;7:70.
87	D'Arrigo S, Gavazzi F, Alfei E, Zuffardi O, Montomoli C, Corso B, et al. The Diagnostic Yield of Array Comparative Genomic Hybridization Is High Regardless of Severity of Intellectual Disability/Developmental Delay in Children. J Child Neurol. 2016;31(6):691-9.
88	D'Gama AM, Mulhern S, Sheidley BR, Boodhoo F, Buts S, Chandler NJ, et al. Evaluation of the feasibility, diagnostic yield, and clinical utility of rapid genome sequencing in infantile epilepsy (Gene-STEPS): an international, multicentre, pilot cohort study. Lancet Neurol. 2023;22(9):812-25.
89	da Cunha Leite AJ, Pinto IP, Leijsten N, Ruiterkamp-Versteeg M, Pfundt R, de Leeuw N, et al. Diagnostic yield of patients with undiagnosed intellectual disability, global developmental delay and multiples congenital anomalies using karyotype, microarray analysis, whole exome sequencing from Central Brazil. PLoS ONE. 2022;17(4 April).

90	Dai L, Zhang D, Wu Z, Guan X, Ma M, Li L, et al. A Tiered Genetic Screening Strategy for the Molecular Diagnosis of Intellectual Disability in Chinese Patients. Front Genet. 2021;12:669217.
91	de Ligt J, Willemsen MH, van Bon BW, Kleefstra T, Yntema HG, Kroes T, et al. Diagnostic exome sequencing in persons with severe intellectual disability. N Engl J Med. 2012;367(20):1921-9.
92	de Souza LC, Dos Santos AP, Sgardioli IC, Viguetti-Campos NL, Marques Prota JR, de Oliveira-Sobrinho RP, et al. Phenotype comparison among individuals with developmental delay/intellectual disability with or without genomic imbalances. J Intellect Disabil Res. 2019;63(11):1379-89.
93	De Wachter M, Schoonjans AS, Weckhuysen S, Van Schil K, Löfgren A, Meuwissen M, et al. From diagnosis to treatment in genetic epilepsies: Implementation of precision medicine in real-world clinical practice. Eur J Paediatr Neurol. 2023;48:46-60.
94	Della Mina E, Ciccone R, Brustia F, Bayindir B, Limongelli I, Vetro A, et al. Improving molecular diagnosis in epilepsy by a dedicated high-throughput sequencing platform. Eur J Hum Genet. 2015;23(3):354-62.
95	Demarest S, Calhoun J, Eschbach K, Yu HC, Mirsky D, Angione K, et al. Whole-exome sequencing and adrenocorticotropic hormone therapy in individuals with infantile spasms. Dev Med Child Neurol. 2022;64(5):633-40.
96	Demos M, Guella I, DeGuzman C, McKenzie MB, Buerki SE, Evans DM, et al. Diagnostic Yield and Treatment Impact of Targeted Exome Sequencing in Early-Onset Epilepsy. Front Neurol. 2019;10:434.
97	Di Gregorio E, Riberi E, Belligni EF, Biamino E, Spielmann M, Ala U, et al. Copy number variants analysis in a cohort of isolated and syndromic developmental delay/intellectual disability reveals novel genomic disorders, position effects and candidate disease genes. Clin Genet. 2017;92(4):415-22.
98	Dimassi S, Labalme A, Ville D, Calender A, Mignot C, Boutry-Kryza N, et al. Whole-exome sequencing improves the diagnosis yield in sporadic infantile spasm syndrome. Clin Genet. 2016;89(2):198-204.
99	Dong X, Liu B, Yang L, Wang H, Wu B, Liu R, et al. Clinical exome sequencing as the first-tier test for diagnosing developmental disorders covering both CNV and SNV: a Chinese cohort. J Med Genet. 2020;57(8):558-66.
100	Du X, An Y, Yu L, Liu R, Qin Y, Guo X, et al. A genomic copy number variant analysis implicates the MBD5 and HNRNPU genes in Chinese children with infantile spasms and expands the clinical spectrum of 2q23.1 deletion. BMC Med Genet. 2014;15:62.
101	Du X, Gao X, Liu X, Shen L, Wang K, Fan Y, et al. Genetic Diagnostic Evaluation of Trio-Based Whole Exome Sequencing Among Children With Diagnosed or Suspected Autism Spectrum Disorder. Front Genet. 2018;9:594.
102	Duc NM, Thu NTM, Bui CB, Hoa G, Le Trung Hieu N. Genotype and phenotype characteristics of West syndrome in 20 Vietnamese children: Two novel variants detected by next-generation sequencing. Epilepsy Res. 2023;190:107094.
103	Dunn PJ, Maher BH, Albury CL, Stuart S, Sutherland HG, Maksemous N, et al. Tiered analysis of whole-exome sequencing for epilepsy diagnosis. Mol Genet Genomics. 2020;295(3):751-63.
104	Duzkale N, Akın R. Frequency of Specific Genes in Different Types of Epilepsy. J Coll Physicians

	Surg Pak. 2021;31(11):1296-302.
105	Espeche LD, Solari AP, Mori M, Arenas RM, Palomares M, Pérez M, et al. Implementation of chromosomal microarrays in a cohort of patients with intellectual disability at the Argentinean public health system. Mol Biol Rep. 2020;47(9):6863-78.
106	Essajee F, Urban M, Smit L, Wilmshurst JM, Solomons R, van Toorn R, et al. Utility of genetic testing in children with developmental and epileptic encephalopathy (DEE) at a tertiary hospital in South Africa: A prospective study. Seizure. 2022;101:197-204.
107	Evers C, Staufner C, Granzow M, Paramasivam N, Hinderhofer K, Kaufmann L, et al. Impact of clinical exomes in neurodevelopmental and neurometabolic disorders. Mol Genet Metab. 2017;121(4):297-307.
108	Ewans LJ, Schofield D, Shrestha R, Zhu Y, Gayevskiy V, Ying K, et al. Whole-exome sequencing reanalysis at 12 months boosts diagnosis and is cost-effective when applied early in Mendelian disorders. Genet Med. 2018;20(12):1564-74.
109	Faheem M, Naseer MI, Chaudhary AG, Kumosani TA, Rasool M, Algahtani HA, et al. Array-comparative genomic hybridization analysis of a cohort of Saudi patients with epilepsy. CNS Neurol Disord Drug Targets. 2015;14(4):468-75.
110	Fan Y, Du X, Liu X, Wang L, Li F, Yu Y. Rare Copy Number Variations in a Chinese Cohort of Autism Spectrum Disorder. Front Genet. 2018;9:665.
111	Fan Y, Wu Y, Wang L, Wang Y, Gong Z, Qiu W, et al. Chromosomal microarray analysis in developmental delay and intellectual disability with comorbid conditions. BMC Med Genomics. 2018;11(1):49.
112	Fernández-Marmiesse A, Roca I, Díaz-Flores F, Cantarín V, Pérez-Poyato MS, Fontalba A, et al. Rare Variants in 48 Genes Account for 42% of Cases of Epilepsy With or Without Neurodevelopmental Delay in 246 Pediatric Patients. Front Neurosci. 2019;13:1135.
113	Firouzabadi SG, Kariminejad R, Vameghi R, Darvish H, Ghaedi H, Banihashemi S, et al. Copy Number Variants in Patients with Autism and Additional Clinical Features: Report of VIPR2 Duplication and a Novel Microduplication Syndrome. Mol Neurobiol. 2017;54(9):7019-27.
114	Francis DI, Stark Z, Scheffer IE, Tan TY, Murali K, Gallacher L, et al. Comparing saliva and blood for the detection of mosaic genomic abnormalities that cause syndromic intellectual disability. Eur J Hum Genet. 2023;31(5):521-5.
115	Fung CW, Kwong AK, Wong VC. Gene panel analysis for nonsyndromic cryptogenic neonatal/infantile epileptic encephalopathy. Epilepsia Open. 2017;2(2):236-43.
116	Galizia EC, Srikantha M, Palmer R, Waters JJ, Lench N, Ogilvie CM, et al. Array comparative genomic hybridization: results from an adult population with drug-resistant epilepsy and co-morbidities. Eur J Med Genet. 2012;55(5):342-8.
117	Gall K, Izzo E, Seppälä EH, Alakurtti K, Koskinen L, Saarinen I, et al. Next-generation sequencing in childhood-onset epilepsies: Diagnostic yield and impact on neuronal ceroid lipofuscinosis type 2 (CLN2) disease diagnosis. PLoS One. 2021;16(9):e0255933.
118	Gao C, Wang X, Mei S, Li D, Duan J, Zhang P, et al. Diagnostic Yields of Trio-WES Accompanied by CNVseq for Rare Neurodevelopmental Disorders. Front Genet. 2019;10:485.
119	Garrido-Torres N, Marqués Rodríguez R, Alemany-Navarro M, Sánchez-García J, García-Cerro S, Ayuso MI, et al. Exploring genetic testing requests, genetic alterations and clinical associations in a

	cohort of children with autism spectrum disorder. Eur Child Adolesc Psychiatry. 2024.
120	Gerik-Celebi HB, Aydin H, Bolat H, Unsel-Bolat G. Clinical and Genetic Characteristics of Patients with Unexplained Intellectual Disability/Developmental Delay without Epilepsy. Mol Syndromol. 2023;14(3):208-18.
121	Gieldon L, Mackenroth L, Kahlert AK, Lemke JR, Porrmann J, Schallner J, et al. Diagnostic value of partial exome sequencing in developmental disorders. PLoS One. 2018;13(8):e0201041.
122	Gilissen C, Hehir-Kwa JY, Thung DT, van de Vorst M, van Bon BW, Willemsen MH, et al. Genome sequencing identifies major causes of severe intellectual disability. Nature. 2014;511(7509):344-7.
123	Gokben S, Onay H, Yilmaz S, Atik T, Serdaroglu G, Tekin H, et al. Targeted next generation sequencing: the diagnostic value in early-onset epileptic encephalopathy. Acta Neurol Belg. 2017;117(1):131-8.
124	Graifman JL, Lippa NC, Mulhern MS, Bergner AL, Sands TT. Clinical utility of exome sequencing in a pediatric epilepsy cohort. Epilepsia. 2023;64(4):986-97.
125	Grether A, Ivanovski I, Russo M, Begemann A, Steindl K, Abela L, et al. The current benefit of genome sequencing compared to exome sequencing in patients with developmental or epileptic encephalopathies. Mol Genet Genomic Med. 2023:e2148.
126	Grozeva D, Carss K, Spasic-Boskovic O, Tejada MI, Gecz J, Shaw M, et al. Targeted Next-Generation Sequencing Analysis of 1,000 Individuals with Intellectual Disability. Hum Mutat. 2015;36(12):1197-204.
127	Gun-Bilgic D, Polat M. Analysis of the Pathogenic Variants of Genes Using a Gene Panel in Turkish Epilepsy Patients. Clin Lab. 2022;68(6).
128	Guo MH, Bardakjian TM, Brzozowski MR, Scherer SS, Quinn C, Elman L, et al. Temporal trends and yield of clinical diagnostic genetic testing in adult neurology. Am J Med Genet A. 2021;185(10):2922-8.
129	Gürkan H, Atli E, Atli E, Bozatli L, Altay MA, Yalçintepe S, et al. Chromosomal Microarray Analysis in Turkish Patients with Unexplained Developmental Delay and Intellectual Developmental Disorders. Noro Psikiyatr Ars. 2020;57(3):177-91.
130	Halvardson J, Zhao JJ, Zaghlool A, Wentzel C, Georgii-Hemming P, Månsson E, et al. Mutations in HECW2 are associated with intellectual disability and epilepsy. J Med Genet. 2016;53(10):697-704.
131	Hamdan FF, Myers CT, Cossette P, Lemay P, Spiegelman D, Laporte AD, et al. High Rate of Recurrent De Novo Mutations in Developmental and Epileptic Encephalopathies. Am J Hum Genet. 2017;101(5):664-85.
132	Han JY, Jang JH, Park J, Lee IG. Targeted Next-Generation Sequencing of Korean Patients With Developmental Delay and/or Intellectual Disability. Front Pediatr. 2018;6:391.
133	Han JY, Jang W, Park J, Kim M, Kim Y, Lee IG. Diagnostic approach with genetic tests for global developmental delay and/or intellectual disability: Single tertiary center experience. Ann Hum Genet. 2019;83(3):115-23.
134	Haviland I, Daniels CI, Greene CA, Drew J, Love-Nichols JA, Swanson LC, et al. Genetic Diagnosis Impacts Medical Management for Pediatric Epilepsies. Pediatr Neurol. 2023;138:71-80.
135	Helbig KL, Farwell Hagman KD, Shinde DN, Mroske C, Powis Z, Li S, et al. Diagnostic exome sequencing provides a molecular diagnosis for a significant proportion of patients with epilepsy.

	Genet Med. 2016;18(9):898-905.
136	Hesse AN, Bevilacqua J, Shankar K, Reddi HV. Retrospective genotype-phenotype analysis in a 305 patient cohort referred for testing of a targeted epilepsy panel. Epilepsy Res. 2018;144:53-61.
137	Hildebrand MS, Myers CT, Carvill GL, Regan BM, Damiano JA, Mullen SA, et al. A targeted resequencing gene panel for focal epilepsy. Neurology. 2016;86(17):1605-12.
138	Hino-Fukuyo N, Kikuchi A, Arai-Ichinoi N, Niihori T, Sato R, Suzuki T, et al. Genomic analysis identifies candidate pathogenic variants in 9 of 18 patients with unexplained West syndrome. Hum Genet. 2015;134(6):649-58.
139	Hiraide T, Yamoto K, Masunaga Y, Asahina M, Endoh Y, Ohkubo Y, et al. Genetic and phenotypic analysis of 101 patients with developmental delay or intellectual disability using whole-exome sequencing. Clin Genet. 2021;100(1):40-50.
140	Hiz Kurul S, Oktay Y, Töpf A, Szabó NZ, Güngör S, Yaramis A, et al. High diagnostic rate of trio exome sequencing in consanguineous families with neurogenetic diseases. Brain. 2022;145(4):1507-18.
141	Hnoonual A, Thammachote W, Tim-Aroon T, Rojnueangnit K, Hansakunachai T, Sombuntham T, et al. Chromosomal microarray analysis in a cohort of underrepresented population identifies SERINC2 as a novel candidate gene for autism spectrum disorder. Sci Rep. 2017;7(1):12096.
142	Ho KS, Twede H, Vanzo R, Harward E, Hensel CH, Martin MM, et al. Clinical Performance of an Ultrahigh Resolution Chromosomal Microarray Optimized for Neurodevelopmental Disorders. Biomed Res Int. 2016;2016:3284534.
143	Ho KS, Wassman ER, Baxter AL, Hensel CH, Martin MM, Prasad A, et al. Chromosomal Microarray Analysis of Consecutive Individuals with Autism Spectrum Disorders Using an Ultra-High Resolution Chromosomal Microarray Optimized for Neurodevelopmental Disorders. Int J Mol Sci. 2016;17(12).
144	Hoelz H, Herdl C, Gerstl L, Tacke M, Vill K, von Stuelpnagel C, et al. Impact on Clinical Decision Making of Next-Generation Sequencing in Pediatric Epilepsy in a Tertiary Epilepsy Referral Center. Clin EEG Neurosci. 2020;51(1):61-9.
145	Horák O, Burešová M, Kolář S, Španělová K, Jeřábková B, Gaillyová R, et al. Next-generation sequencing in children with epilepsy: The importance of precise genotype-phenotype correlation. Epilepsy Behav. 2022;128:108564.
146	Howell KB, Eggers S, Dalziel K, Riseley J, Mandelstam S, Myers CT, et al. A population-based cost-effectiveness study of early genetic testing in severe epilepsies of infancy. Epilepsia. 2018;59(6):1177-87.
147	Howell KB, Kornberg AJ, Harvey AS, Ryan MM, Mackay MT, Freeman JL, et al. High resolution chromosomal microarray in undiagnosed neurological disorders. J Paediatr Child Health. 2013;49(9):716-24.
148	Hsu RH, Lee CH, Chien YH, Lin SP, Hung MZ, Chen NC, et al. Utility of whole-exome sequencing for patients with multiple congenital anomalies with or without intellectual disability/developmental delay in East Asia population. Mol Genet Genomic Med. 2023;11(6):e2160.
149	Hu C, Wang Y, Li C, Mei L, Zhou B, Li D, et al. Targeted sequencing and clinical strategies in children with autism spectrum disorder: A cohort study. Front Genet. 2023;14:1083779.
150	Hu T, Zhang Z, Wang J, Li Q, Zhu H, Lai Y, et al. Chromosomal Aberrations in Pediatric Patients

	with Developmental Delay/Intellectual Disability: A Single-Center Clinical Investigation. Biomed Res Int. 2019;2019:9352581.
151	Ibarluzea N, Hoz AB, Villate O, Llano I, Ocio I, Martí I, et al. Targeted Next-Generation Sequencing in Patients with Suggestive X-Linked Intellectual Disability. Genes (Basel). 2020;11(1).
152	Iourov IY, Vorsanova SG, Kurinnaia OS, Zelenova MA, Silvanovich AP, Yurov YB. Molecular karyotyping by array CGH in a Russian cohort of children with intellectual disability, autism, epilepsy and congenital anomalies. Mol Cytogenet. 2012;5(1):46.
153	Ishay RTB, Shil A, Solomon S, Sadigurschi N, Abu-Kaf H, Meiri G, et al. Diagnostic yield and economic implications of whole-exome sequencing for asd diagnosis in israel. Genes. 2022;13(1).
154	Jalkh N, Corbani S, Haidar Z, Hamdan N, Farah E, Abou Ghoch J, et al. The added value of WES reanalysis in the field of genetic diagnosis: lessons learned from 200 exomes in the Lebanese population. BMC Med Genomics. 2019;12(1):11.
155	Jang SS, Kim SY, Kim H, Hwang H, Chae JH, Kim KJ, et al. Diagnostic Yield of Epilepsy Panel Testing in Patients With Seizure Onset Within the First Year of Life. Front Neurol. 2019;10:988.
156	Jang W, Kim Y, Han E, Park J, Chae H, Kwon A, et al. Chromosomal Microarray Analysis as a First-Tier Clinical Diagnostic Test in Patients With Developmental Delay/Intellectual Disability, Autism Spectrum Disorders, and Multiple Congenital Anomalies: A Prospective Multicenter Study in Korea. Ann Lab Med. 2019;39(3):299-310.
157	Jezela-Stanek A, Ciara E, Jurkiewicz D, Kucharczyk M, Jędrzejowska M, Chrzanowska KH, et al. The phenotype-driven computational analysis yields clinical diagnosis for patients with atypical manifestations of known intellectual disability syndromes. Mol Genet Genomic Med. 2020;8(9):e1263.
158	Jiang YL, Song C, Wang Y, Zhao J, Yang F, Gao Q, et al. Clinical Utility of Exome Sequencing and Reinterpreting Genetic Test Results in Children and Adults With Epilepsy. Front Genet. 2020;11:591434.
159	Jiao Q, Sun H, Zhang H, Wang R, Li S, Sun D, et al. The combination of whole-exome sequencing and copy number variation sequencing enables the diagnosis of rare neurological disorders. Clin Genet. 2019;96(2):140-50.
160	João S, Quental R, Pinto J, Almeida C, Santos H, Dória S. Impact of copy number variants in epilepsy plus neurodevelopment disorders. Seizure. 2024;117:6-12.
161	Johannesen KM, Nikanorova N, Marjanovic D, Pavbro A, Larsen LHG, Rubboli G, et al. Utility of genetic testing for therapeutic decision-making in adults with epilepsy. Epilepsia. 2020;61(6):1234-9.
162	Joly G, Lapierre JM, Ozilou C, Gosset P, Aurias A, de Blois MC, et al. Comparative genomic hybridisation in mentally retarded patients with dysmorphic features and a normal karyotype. Clin Genet. 2001;60(3):212-9.
163	Jose M, Fasaludeen A, Pavuluri H, Rudrabhatla PK, Chandrasekharan SV, Jose J, et al. Metabolic causes of pediatric developmental & epileptic encephalopathies (DEE)- genetic variant analysis in a south Indian cohort. Seizure. 2024;115:20-7.
164	Kahrizi K, Hu H, Hosseini M, Kalscheuer VM, Fattahi Z, Beheshtian M, et al. Effect of inbreeding on intellectual disability revisited by trio sequencing. Clin Genet. 2019;95(1):151-9.
165	Kalsner L, Twachtman-Bassett J, Tokarski K, Stanley C, Dumont-Mathieu T, Cotney J, et al.

	Genetic testing including targeted gene panel in a diverse clinical population of children with autism spectrum disorder: Findings and implications. Mol Genet Genomic Med. 2018;6(2):171-85.
166	Kamath V, Yoganathan S, Thomas MM, Gowri M, Chacko MP. Utility of Chromosomal Microarray in Children with Unexplained Developmental Delay/Intellectual Disability. Fetal Pediatr Pathol. 2022;41(2):208-18.
167	Kang KW, Kim W, Cho YW, Lee SK, Jung KY, Shin W, et al. Genetic characteristics of non-familial epilepsy. PeerJ. 2019;7:e8278.
168	Kanmaz S, Yılmaz S, Olculu CB, Toprak DE, Ince T, Yılmaz Ö, et al. The Utility of Genetic Testing in Infantile Epileptic Spasms Syndrome: A Step-Based Approach in the Next-Generation Sequencing Era. Pediatr Neurol. 2024;157:100-7.
169	Kashevarova AA, Nazarenko LP, Skryabin NA, Salyukova OA, Chechetkina NN, Tolmacheva EN, et al. Array CGH analysis of a cohort of Russian patients with intellectual disability. Gene. 2014;536(1):145-50.
170	Kim J, Lee J, Jang DH. Combining chromosomal microarray and clinical exome sequencing for genetic diagnosis of intellectual disability. Sci Rep. 2023;13(1):22807.
171	Kim J, Lee J, Kim M, Jang DH. Diagnostic Yield of Trio Whole-Genome Sequencing in Children with Undiagnosed Developmental Delay or Congenital Anomaly: A Prospective Cohort Study. Diagnostics (Basel). 2024;14(15).
172	Kim SH, Kim B, Lee JS, Kim HD, Choi JR, Lee ST, et al. Proband-Only Clinical Exome Sequencing for Neurodevelopmental Disabilities. Pediatr Neurol. 2019;99:47-54.
173	Kim SH, Seo J, Kwon SS, Teng LY, Won D, Shin S, et al. Common genes and recurrent causative variants in 957 Asian patients with pediatric epilepsy. Epilepsia. 2023.
174	Kirchhoff M, Pedersen S, Kjeldsen E, Rose H, Dunø M, Kølvraa S, et al. Prospective study comparing HR-CGH and subtelomeric FISH for investigation of individuals with mental retardation and dysmorphic features and an update of a study using only HR-CGH. Am J Med Genet A. 2004;127a(2):111-7.
175	Ko A, Youn SE, Kim SH, Lee JS, Kim S, Choi JR, et al. Targeted gene panel and genotype-phenotype correlation in children with developmental and epileptic encephalopathy. Epilepsy Res. 2018;141:48-55.
176	Kocaaga A, Yimenicioglu S. Identification of Novel Gene Variants in Children With Drug-Resistant Epilepsy: Expanding the Genetic Spectrum. Pediatr Neurol. 2023;139:7-12.
177	Kodera H, Kato M, Nord AS, Walsh T, Lee M, Yamanaka G, et al. Targeted capture and sequencing for detection of mutations causing early onset epileptic encephalopathy. Epilepsia. 2013;54(7):1262-9.
178	Koh HY, Smith L, Wiltrout KN, Podury A, Chourasia N, D'Gama AM, et al. Utility of Exome Sequencing for Diagnosis in Unexplained Pediatric-Onset Epilepsy. JAMA Netw Open. 2023;6(7):e2324380.
179	Kousoulidou L, Moutafi M, Nicolaides P, Hadjiloizou S, Christofi C, Paradesiotou A, et al. Screening of 50 cypriot patients with autism spectrum disorders or autistic features using 400K custom array-CGH. Biomed Res Int. 2013;2013:843027.
180	Krenn M, Wagner M, Hotzy C, Graf E, Weber S, Brunet T, et al. Diagnostic exome sequencing in non-acquired focal epilepsies highlights a major role of GATOR1 complex genes. J Med Genet.

	2020;57(9):624-33.
181	Krepischi ACV, Villela D, da Costa SS, Mazzonetto PC, Schauren J, Migliavacca MP, et al. Chromosomal microarray analyses from 5778 patients with neurodevelopmental disorders and congenital anomalies in Brazil. Sci Rep. 2022;12(1):15184.
182	Krey I, Krois-Neudenberger J, Hentschel J, Syrbe S, Polster T, Hanker B, et al. Genotype-phenotype correlation on 45 individuals with West syndrome. Eur J Paediatr Neurol. 2020;25:134-8.
183	Krgovic D, Gorenjak M, Rihar N, Opalic I, Stangler Herodez S, Gregoric Kumperscak H, et al. Impaired Neurodevelopmental Genes in Slovenian Autistic Children Elucidate the Comorbidity of Autism With Other Developmental Disorders. Front Mol Neurosci. 2022;15:912671.
184	Krygier M, Pietruszka M, Zawadzka M, Sawicka A, Lemska A, Limanówka M, et al. Next-generation sequencing testing in children with epilepsy reveals novel clinical, diagnostic and therapeutic implications. Front Genet. 2023;14:1300952.
185	Lee CG, Lee J, Lee M. Multi-gene panel testing in Korean patients with common genetic generalized epilepsy syndromes. PLoS One. 2018;13(6):e0199321.
186	Lee CG, Park SJ, Yun JN, Ko JM, Kim HJ, Yim SY, et al. Array-based comparative genomic hybridization in 190 Korean patients with developmental delay and/or intellectual disability: a single tertiary care university center study. Yonsei Med J. 2013;54(6):1463-70.
187	Lee CL, Chuang CK, Tu RY, Chiu HC, Lo YT, Chang YH, et al. Increased Diagnostic Yield of Array Comparative Genomic Hybridization for Autism Spectrum Disorder in One Institution in Taiwan. Medicina (Kaunas). 2021;58(1).
188	Lee CL, Lee CH, Chuang CK, Chiu HC, Chen YJ, Chou CL, et al. Array-CGH increased the diagnostic rate of developmental delay or intellectual disability in Taiwan. Pediatr Neonatol. 2019;60(4):453-60.
189	Lee HF, Chi CS, Tsai CR. Diagnostic yield and treatment impact of whole-genome sequencing in paediatric neurological disorders. Dev Med Child Neurol. 2021;63(8):934-8.
190	Lee J, Ha S, Lee ST, Park SG, Shin S, Choi JR, et al. Next-Generation Sequencing in Korean Children With Autism Spectrum Disorder and Comorbid Epilepsy. Front Pharmacol. 2020;11:585.
191	Lee J, Lee C, Ki CS, Lee J. Determining the best candidates for next-generation sequencing-based gene panel for evaluation of early-onset epilepsy. Mol Genet Genomic Med. 2020;8(9):e1376.
192	Lee J, Lee C, Park WY, Lee J. Genetic Diagnosis of Dravet Syndrome Using Next Generation Sequencing-Based Epilepsy Gene Panel Testing. Annals of Clinical and Laboratory Science. 2020;50(5):625-37.
193	Lee JS, Hwang H, Kim SY, Kim KJ, Choi JS, Woo MJ, et al. Chromosomal Microarray With Clinical Diagnostic Utility in Children With Developmental Delay or Intellectual Disability. Ann Lab Med. 2018;38(5):473-80.
194	Lee KY, Shin E. Application of array comparative genomic hybridization in Korean children under 6 years old with global developmental delay. Korean J Pediatr. 2017;60(9):282-9.
195	Lee S, Kang MK, So KH, Jang R, Shin YW, Jang SS, et al. Broadening the scope of multigene panel analysis for adult epilepsy patients. Epilepsia Open. 2024;9(4):1538-49.
196	Lee S, Karp N, Zapata-Aldana E, Sadikovic B, Yang P, Balci TB, et al. Genetic Testing in Children with Epilepsy: Report of a Single-Center Experience. Can J Neurol Sci. 2021;48(2):233-44.

197	Lee SH, Song WJ. Chromosomal Microarray Testing in 42 Korean Patients with Unexplained Developmental Delay, Intellectual Disability, Autism Spectrum Disorders, and Multiple Congenital Anomalies. Genomics Inform. 2017;15(3):82-6.
198	Lee Z, Lee BJ, Park S, Park D. Relationship between Clinical Parameters and Chromosomal Microarray Data in Infants with Developmental Delay. Healthcare (Basel). 2020;8(3).
199	Leite A, Pinto IP, Leijsten N, Ruiterkamp-Versteeg M, Pfundt R, de Leeuw N, et al. Diagnostic yield of patients with undiagnosed intellectual disability, global developmental delay and multiples congenital anomalies using karyotype, microarray analysis, whole exome sequencing from Central Brazil. PLoS One. 2022;17(4):e0266493.
200	Lemke JR, Riesch E, Scheurenbrand T, Schubach M, Wilhelm C, Steiner I, et al. Targeted next generation sequencing as a diagnostic tool in epileptic disorders. Epilepsia. 2012;53(8):1387-98.
201	Lengyel A, Pinti É, Pikó H, Kristóf Á, Abonyi T, Némethi Z, et al. Clinical evaluation of rare copy number variations identified by chromosomal microarray in a Hungarian neurodevelopmental disorder patient cohort. Mol Cytogenet. 2022;15(1):47.
202	Li J, Gao K, Yan H, Xiangwei W, Liu N, Wang T, et al. Reanalysis of whole exome sequencing data in patients with epilepsy and intellectual disability/mental retardation. Gene. 2019;700:168-75.
203	Li Y, Lei D, Li C, Huang D, Tan J, Zhang H. [Diagnostic value of whole exome sequencing for patients with intellectual disability or global developmental delay]. Zhonghua Yi Xue Yi Chuan Xue Za Zhi. 2023;40(6):648-54.
204	Li Y, Qiu W, Ye J, Han L, Zhang H, Gu X. [Analysis of copy number variations in 66 children with unexplained mental retardation/developmental delay using chromosomal microarrays]. Zhonghua Yi Xue Yi Chuan Xue Za Zhi. 2014;31(6):703-7.
205	Licchetta L, Pippucci T, Baldassari S, Minardi R, Provini F, Mostacci B, et al. Sleep-related hypermotor epilepsy (SHE): Contribution of known genes in 103 patients. Seizure. 2020;74:60-4.
206	Lin ZJ, Li B, Lin PX, Song W, Yan LM, Meng H, et al. Clinical application of trio-based whole-exome sequencing in idiopathic generalized epilepsy. Seizure. 2023.
207	Lindstrand A, Eisfeldt J, Pettersson M, Carvalho CMB, Kvarnung M, Grigelioniene G, et al. From cytogenetics to cytogenomics: whole-genome sequencing as a first-line test comprehensively captures the diverse spectrum of disease-causing genetic variation underlying intellectual disability. Genome Med. 2019;11(1):68.
208	Lindstrand A, Ek M, Kvarnung M, Anderlid BM, Björck E, Carlsten J, et al. Genome sequencing is a sensitive first-line test to diagnose individuals with intellectual disability. Genet Med. 2022;24(11):2296-307.
209	Lintas C, Picinelli C, Piras IS, Sacco R, Brogna C, Persico AM. Copy number variation in 19 Italian multiplex families with autism spectrum disorder: Importance of synaptic and neurite elongation genes. Am J Med Genet B Neuropsychiatr Genet. 2017;174(5):547-56.
210	Liu J, Tong L, Song S, Niu Y, Li J, Wu X, et al. Novel and de novo mutations in pediatric refractory epilepsy. Mol Brain. 2018;11(1):48.
211	Liu X, Shen Q, Zheng G, Guo H, Lu X, Wang X, et al. Gene and Phenotype Expansion of Unexplained Early Infantile Epileptic Encephalopathy. Front Neurol. 2021;12:633637.
212	Liu Y, Lv Y, Zarrei M, Dong R, Yang X, Higginbotham EJ, et al. Chromosomal microarray analysis of 410 Han Chinese patients with autism spectrum disorder or unexplained intellectual disability

	and developmental delay. NPJ Genom Med. 2022;7(1):1.
213	Lob K, Sawka DM, Gaitanis JN, Liu JS, Nie DA. Genetic Diagnostic Yield in Autism Spectrum Disorder (ASD) and Epilepsy Phenotypes in Children with Genetically Defined ASD. J Autism Dev Disord. 2024.
214	Long S, Zhou H, Li S, Wang T, Ma Y, Li C, et al. The Clinical and Genetic Features of Co-occurring Epilepsy and Autism Spectrum Disorder in Chinese Children. Front Neurol. 2019;10:505.
215	Lopes F, Torres F, Soares G, Barbosa M, Silva J, Duque F, et al. Genomic imbalances defining novel intellectual disability associated loci. Orphanet J Rare Dis. 2019;14(1):164.
216	Lovrečić L, Rajar P, Volk M, Bertok S, Gnidovec Stražišar B, Osredkar D, et al. Diagnostic efficacy and new variants in isolated and complex autism spectrum disorder using molecular karyotyping. J Appl Genet. 2018;59(2):179-85.
217	Lowther C, Valkanas E, Giordano JL, Wang HZ, Currall BB, O'Keefe K, et al. Systematic evaluation of genome sequencing for the diagnostic assessment of autism spectrum disorder and fetal structural anomalies. Am J Hum Genet. 2023;110(9):1454-69.
218	Lumaka A, Race V, Peeters H, Corveleyn A, Coban-Akdemir Z, Jhangiani SN, et al. A comprehensive clinical and genetic study in 127 patients with ID in Kinshasa, DR Congo. American Journal of Medical Genetics, Part A. 2018;176(9):1897-909.
219	Lund C, Brodtkorb E, Røsby O, Rødningen OK, Selmer KK. Copy number variants in adult patients with Lennox-Gastaut syndrome features. Epilepsy Res. 2013;105(1-2):110-7.
220	Ma H, Zhu L, Yang X, Ao M, Zhang S, Guo M, et al. Genetic and phenotypic analysis of 225 Chinese children with developmental delay and/or intellectual disability using whole-exome sequencing. BMC Genomics. 2024;25(1):391.
221	Mainali A, Athey T, Bahl S, Hung C, Caluseriu O, Chan A, et al. Diagnostic yield of clinical exome sequencing in adulthood in medical genetics clinics. Am J Med Genet A. 2023;191(2):510-7.
222	Maini I, Ivanovski I, Djuric O, Caraffi SG, Errichiello E, Marinelli M, et al. Prematurity, ventricular septal defect and dysmorphisms are independent predictors of pathogenic copy number variants: a retrospective study on array-CGH results and phenotypical features of 293 children with neurodevelopmental disorders and/or multiple congenital anomalies. Ital J Pediatr. 2018;44(1):34.
223	Mak ASL, Chiu ATG, Leung GKC, Mak CCY, Chu YWY, Mok GTK, et al. Use of clinical chromosomal microarray in Chinese patients with autism spectrum disorder-implications of a copy number variation involving DPP10. Mol Autism. 2017;8:31.
224	Manolakos E, Vetro A, Kefalas K, Rapti SM, Louizou E, Garas A, et al. The use of array-CGH in a cohort of Greek children with developmental delay. Mol Cytogenet. 2010;3:22.
225	Martínez F, Caro-Llopis A, Roselló M, Oltra S, Mayo S, Monfort S, et al. High diagnostic yield of syndromic intellectual disability by targeted next-generation sequencing. J Med Genet. 2017;54(2):87-92.
226	Martinez-Granero F, Blanco-Kelly F, Sanchez-Jimeno C, Avila-Fernandez A, Arteche A, Bustamante-Aragones A, et al. Comparison of the diagnostic yield of aCGH and genome-wide sequencing across different neurodevelopmental disorders. NPJ Genom Med. 2021;6(1):25.
227	Martucci M, Novelli M, Scarselli V, Di Palma F, Fiorelli F, Bernardini L, et al. Array-comparative genomic hybridization analysis in a cohort of 130 children with Autism Spectrum Disorders: A single Center Italian Study. Clin Ter. 2023;174(6):509-17.

228	Masri AT, Oweis L, Ali M, Hamamy H. Global developmental delay and intellectual disability in the era of genomics: Diagnosis and challenges in resource limited areas. Clin Neurol Neurosurg. 2023;230:107799.
229	McGrew SG, Peters BR, Crittendon JA, Veenstra-Vanderweele J. Diagnostic yield of chromosomal microarray analysis in an autism primary care practice: which guidelines to implement? J Autism Dev Disord. 2012;42(8):1582-91.
230	McKnight D, Bristow SL, Truty RM, Morales A, Stetler M, Westbrook MJ, et al. Multigene Panel Testing in a Large Cohort of Adults With Epilepsy: Diagnostic Yield and Clinically Actionable Genetic Findings. Neurol Genet. 2022;8(1):e650.
231	Megahed H, Nicouleau M, Barcia G, Medina-Cano D, Siquier-Pernet K, Bole-Feysot C, et al. Utility of whole exome sequencing for the early diagnosis of pediatric-onset cerebellar atrophy associated with developmental delay in an inbred population. Orphanet J Rare Dis. 2016;11(1):57.
232	Mellone S, Puricelli C, Vurchio D, Ronzani S, Favini S, Maruzzi A, et al. The Usefulness of a Targeted Next Generation Sequencing Gene Panel in Providing Molecular Diagnosis to Patients With a Broad Spectrum of Neurodevelopmental Disorders. Front Genet. 2022;13:875182.
233	Mercimek-Mahmutoglu S, Patel J, Cordeiro D, Hewson S, Callen D, Donner EJ, et al. Diagnostic yield of genetic testing in epileptic encephalopathy in childhood. Epilepsia. 2015;56(5):707-16.
234	Miao P, Feng J, Guo Y, Wang J, Xu X, Wang Y, et al. Genotype and phenotype analysis using an epilepsy-associated gene panel in Chinese pediatric epilepsy patients. Clin Genet. 2018;94(6):512-20.
235	Michaud JL, Lachance M, Hamdan FF, Carmant L, Lortie A, Diadori P, et al. The genetic landscape of infantile spasms. Hum Mol Genet. 2014;23(18):4846-58.
236	Milone R, Cesario C, Goldoni M, Pasquariello R, Fusilli C, Giovannetti A, et al. Correlating Neuroimaging and CNVs Data: 7 Years of Cytogenomic Microarray Analysis on Patients Affected by Neurodevelopmental Disorders. J Pediatr Genet. 2021;10(4):292-9.
237	Mina ED, Ciccone R, Brustia F, Bayindir B, Limongelli I, Vetro A, et al. Improving molecular diagnosis in epilepsy by a dedicated high-throughput sequencing platform. European Journal of Human Genetics. 2015;23(3):354-62.
238	Minardi R, Licchetta L, Baroni MC, Pippucci T, Stipa C, Mostacci B, et al. Whole-exome sequencing in adult patients with developmental and epileptic encephalopathy: It is never too late. Clin Genet. 2020;98(5):477-85.
239	Mir A, AlQahtani M, Amer F, AlBaradie R, AlOtaibi W, AlGhamdi F, et al. Diagnostic evaluation of patients with epileptic spasms in the era of next-generation sequencing. Epileptic Disord. 2024.
240	Mitani T, Isikay S, Gezdirici A, Gulec EY, Punetha J, Fatih JM, et al. High prevalence of multilocus pathogenic variation in neurodevelopmental disorders in the Turkish population. Am J Hum Genet. 2021;108(10):1981-2005.
241	Mitta N, Menon RN, McTague A, Radhakrishnan A, Sundaram S, Cherian A, et al. Genotype-phenotype correlates of infantile-onset developmental & epileptic encephalopathy syndromes in South India: A single centre experience. Epilepsy Res. 2020;166:106398.
242	Moeschler JB, Amato RS, Brewster T, Burke L, Dinulos MB, Smith R, et al. Improving genetic health care: a Northern New England pilot project addressing the genetic evaluation of the child with developmental delays or intellectual disability. Am J Med Genet C Semin Med Genet.

	2009;151c(3):241-54.
243	Moirangthem A, Mandal K, Saxena D, Srivastava P, Gambhir PS, Agrawal N, et al. Genetic heterogeneity of disorders with overgrowth and intellectual disability: Experience from a center in North India. Am J Med Genet A. 2021;185(8):2345-55.
244	Møller RS, Larsen LH, Johannesen KM, Talvik I, Talvik T, Vaher U, et al. Gene Panel Testing in Epileptic Encephalopathies and Familial Epilepsies. Mol Syndromol. 2016;7(4):210-9.
245	Monroe GR, Frederix GW, Savelberg SM, de Vries TI, Duran KJ, van der Smagt JJ, et al. Effectiveness of whole-exome sequencing and costs of the traditional diagnostic trajectory in children with intellectual disability. Genet Med. 2016;18(9):949-56.
246	Mordaunt D, Gabbett M, Waugh M, O'Brien K, Heussler H. Uptake and Diagnostic Yield of Chromosomal Microarray in an Australian Child Development Clinic. Children (Basel). 2014;1(1):21-30.
247	Morgan A, Gandin I, Belcaro C, Palumbo P, Palumbo O, Biamino E, et al. Target sequencing approach intended to discover new mutations in non-syndromic intellectual disability. Mutat Res. 2015;781:32-6.
248	Muir AM, Myers CT, Nguyen NT, Saykally J, Craiu D, De Jonghe P, et al. Genetic heterogeneity in infantile spasms. Epilepsy Res. 2019;156:106181.
249	Mullen SA, Carvill GL, Bellows S, Bayly MA, Trucks H, Lal D, et al. Copy number variants are frequent in genetic generalized epilepsy with intellectual disability. Neurology. 2013;81(17):1507-14.
250	Munnich A, Demily C, Frugère L, Duwime C, Malan V, Barcia G, et al. Impact of on-site clinical genetics consultations on diagnostic rate in children and young adults with autism spectrum disorder. Mol Autism. 2019;10:33.
251	Muona M, Berkovic SF, Dibbens LM, Oliver KL, Maljevic S, Bayly MA, et al. A recurrent de novo mutation in KCNC1 causes progressive myoclonus epilepsy. Nat Genet. 2015;47(1):39-46.
252	Musante L, Costa P, Zanus C, Faletra F, Murru FM, Bianco AM, et al. The Genetic Diagnosis of Ultrarare DEEs: An Ongoing Challenge. Genes (Basel). 2022;13(3).
253	Na JH, Shin S, Yang D, Kim B, Kim HD, Kim S, et al. Targeted gene panel sequencing in early infantile onset developmental and epileptic encephalopathy. Brain Dev. 2020;42(6):438-48.
254	Nambot S, Thevenon J, Kuentz P, Duffourd Y, Tisserant E, Bruel AL, et al. Clinical whole-exome sequencing for the diagnosis of rare disorders with congenital anomalies and/or intellectual disability: substantial interest of prospective annual reanalysis. Genet Med. 2018;20(6):645-54.
255	Napoli E, Russo S, Casula L, Alesi V, Amendola FA, Angioni A, et al. Array-CGH Analysis in a Cohort of Phenotypically Well-Characterized Individuals with "Essential" Autism Spectrum Disorders. J Autism Dev Disord. 2018;48(2):442-9.
256	Naseer MI, Faheem M, Chaudhary AG, Kumosani TA, Al-Quaiti MM, Jan MM, et al. Genome wide analysis of novel copy number variations duplications/deletions of different epileptic patients in Saudi Arabia. BMC Genomics. 2015;16 Suppl 1(Suppl 1):S10.
257	Nassir N, Sati I, Al Shaibani S, Ahmed A, Almidani O, Akter H, et al. Detection of copy number variants and genes by chromosomal microarray in an Emirati neurodevelopmental disorders cohort. Neurogenetics. 2022;23(2):137-49.
258	Newman WG, Hamilton S, Ayres J, Sanghera N, Smith A, Gaunt L, et al. Array comparative

	genomic hybridization for diagnosis of developmental delay: an exploratory cost-consequences analysis. Clin Genet. 2007;71(3):254-9.
259	Nicholl J, Waters W, Mulley JC, Suwalski S, Brown S, Hull Y, et al. Cognitive deficit and autism spectrum disorders: prospective diagnosis by array CGH. Pathology. 2014;46(1):41-5.
260	Numis AL, da Gente G, Sherr EH, Glass HC. Whole-exome sequencing with targeted analysis and epilepsy after acute symptomatic neonatal seizures. Pediatr Res. 2022;91(4):896-902.
261	Oates S, Tang S, Rosch R, Lear R, Hughes EF, Williams RE, et al. Incorporating epilepsy genetics into clinical practice: a 360°evaluation. NPJ Genom Med. 2018;3:13.
262	Ohashi K, Fukuhara S, Miyachi T, Asai T, Imaeda M, Goto M, et al. Comprehensive Genetic Analysis of Non-syndromic Autism Spectrum Disorder in Clinical Settings. J Autism Dev Disord. 2021;51(12):4655-62.
263	Oikonomakis V, Kosma K, Mitrakos A, Sofocleous C, Pervanidou P, Syrmou A, et al. Recurrent copy number variations as risk factors for autism spectrum disorders: analysis of the clinical implications. Clin Genet. 2016;89(6):708-18.
264	Olson H, Shen Y, Avallone J, Sheidley BR, Pinsky R, Bergin AM, et al. Copy number variation plays an important role in clinical epilepsy. Ann Neurol. 2014;75(6):943-58.
265	Ortega-Moreno L, Giráldez BG, Soto-Insuga V, Losada-Del Pozo R, Rodrigo-Moreno M, Alarcón-Morcillo C, et al. Molecular diagnosis of patients with epilepsy and developmental delay using a customized panel of epilepsy genes. PLoS One. 2017;12(11):e0188978.
266	Ostrander BEP, Butterfield RJ, Pedersen BS, Farrell AJ, Layer RM, Ward A, et al. Whole-genome analysis for effective clinical diagnosis and gene discovery in early infantile epileptic encephalopathy. NPJ Genom Med. 2018;3:22.
267	Özaslan A, Kayhan G, İşeri E, Ergün MA, Güney E, Perçin FE. Identification of copy number variants in children and adolescents with autism spectrum disorder: a study from Turkey. Mol Biol Rep. 2021;48(11):7371-8.
268	Palka Bayard de Volo C, Alfonsi M, Morizio E, Guaciali-Franchi P, Mohn A, Chiarelli F. A 343 Italian cohort of patients analysed with array-comparative genomic hybridization: unsolved problems and genetic counselling difficulties. J Intellect Disabil Res. 2021;65(9):863-9.
269	Palmer E, Speirs H, Taylor PJ, Mullan G, Turner G, Einfeld S, et al. Changing interpretation of chromosomal microarray over time in a community cohort with intellectual disability. Am J Med Genet A. 2014;164a(2):377-85.
270	Palmer EE, Sachdev R, Macintosh R, Melo US, Mundlos S, Righetti S, et al. Diagnostic Yield of Whole Genome Sequencing After Nondiagnostic Exome Sequencing or Gene Panel in Developmental and Epileptic Encephalopathies. Neurology. 2021;96(13):e1770-e82.
271	Palmer EE, Schofield D, Shrestha R, Kandula T, Macintosh R, Lawson JA, et al. Integrating exome sequencing into a diagnostic pathway for epileptic encephalopathy: Evidence of clinical utility and cost effectiveness. Mol Genet Genomic Med. 2018;6(2):186-99.
272	Papuc SM, Abela L, Steindl K, Begemann A, Simmons TL, Schmitt B, et al. The role of recessive inheritance in early-onset epileptic encephalopathies: a combined whole-exome sequencing and copy number study. Eur J Hum Genet. 2019;27(3):408-21.
273	Parrini E, Marini C, Mei D, Galuppi A, Cellini E, Pucatti D, et al. Diagnostic Targeted Resequencing in 349 Patients with Drug-Resistant Pediatric Epilepsies Identifies Causative Mutations in 30

	Different Genes. Hum Mutat. 2017;38(2):216-25.
274	Pekeles H, Accogli A, Boudrahem-Addour N, Russell L, Parente F, Srour M. Diagnostic Yield of Intellectual Disability Gene Panels. Pediatr Neurol. 2019;92:32-6.
275	Peltekova I, Yusuf A, Frei J, Savion-Lemieux T, Joober R, Howe J, et al. Predictors of empowerment in parents of children with autism and related neurodevelopmental disorders who are undergoing genetic testing. Mol Genet Genomic Med. 2021;9(11):e1803.
276	Peng J, Pang N, Wang Y, Wang XL, Chen J, Xiong J, et al. Next-generation sequencing improves treatment efficacy and reduces hospitalization in children with drug-resistant epilepsy. CNS Neurosci Ther. 2019;25(1):14-20.
277	Peng J, Wang Y, He F, Chen C, Wu LW, Yang LF, et al. Novel West syndrome candidate genes in a Chinese cohort. CNS Neurosci Ther. 2018;24(12):1196-206.
278	Peng P, Kessi M, Mao L, He F, Zhang C, Chen C, et al. Etiologic Classification of 541 Infantile Spasms Cases: A Cohort Study. Front Pediatr. 2022;10:774828.
279	Pereira RR, Pinto IP, Minasi LB, de Melo AV, da Cruz e Cunha DM, Cruz AS, et al. Screening for intellectual disability using high-resolution CMA technology in a retrospective cohort from Central Brazil. PLoS One. 2014;9(7):e103117.
280	Pereira SSS, Pinto IP, Santos V, Silva RC, Costa EOA, Cruz ASD, et al. Analysis of parental origin of de novo pathogenic CNVs in patients with intellectual disability. Genet Mol Biol. 2024;47(3):e20230313.
281	Perucca P, Scheffer IE, Harvey AS, James PA, Lunke S, Thorne N, et al. Real-world utility of whole exome sequencing with targeted gene analysis for focal epilepsy. Epilepsy Res. 2017;131:1-8.
282	Peycheva V, Kamenarova K, Ivanova N, Stamatov D, Avdjieva-Tzavella D, Alexandrova I, et al. Chromosomal microarray analysis of Bulgarian patients with epilepsy and intellectual disability. Gene. 2018;667:45-55.
283	Pfundt R, Kwiatkowski K, Roter A, Shukla A, Thorland E, Hockett R, et al. Clinical performance of the CytoScan Dx Assay in diagnosing developmental delay/intellectual disability. Genet Med. 2016;18(2):168-73.
284	Pranav Chand R, Vinit W, Vaidya V, Iyer AS, Shelke M, Aggarwal S, et al. Proband only exome sequencing in 403 Indian children with neurodevelopmental disorders: Diagnostic yield, utility and challenges in a resource-limited setting. Eur J Med Genet. 2023;66(5):104730.
285	Qiao Y, Riendeau N, Koochek M, Liu X, Harvard C, Hildebrand MJ, et al. Phenomic determinants of genomic variation in autism spectrum disorders. J Med Genet. 2009;46(10):680-8.
286	Qin YY, Yao YY, Liu N, Wang B, Liu LJ, Li H, et al. [Whole exome sequencing analysis and prenatal diagnosis in children with neurodevelopmental disorders]. Zhonghua Yu Fang Yi Xue Za Zhi. 2023;57(5):753-9.
287	Quaio C, Moreira CM, Novo-Filho GM, Sacramento-Bobotis PR, Groenner Penna M, Perazzio SF, et al. Diagnostic power and clinical impact of exome sequencing in a cohort of 500 patients with rare diseases. Am J Med Genet C Semin Med Genet. 2020;184(4):955-64.
288	Ream MA, Mikati MA. Clinical utility of genetic testing in pediatric drug-resistant epilepsy: a pilot study. Epilepsy Behav. 2014;37:241-8.
289	Redin C, Gérard B, Lauer J, Herenger Y, Muller J, Quartier A, et al. Efficient strategy for the molecular diagnosis of intellectual disability using targeted high-throughput sequencing. J Med

	Genet. 2014;51(11):724-36.
290	Refeat MM, Naggar WE, Saied MME, Kilany A. Whole exome screening of neurodevelopmental regression disorders in a cohort of Egyptian patients. Neurogenetics. 2023;24(1):17-28.
291	Rim JH, Kim SH, Hwang IS, Kwon SS, Kim J, Kim HW, et al. Efficient strategy for the molecular diagnosis of intractable early-onset epilepsy using targeted gene sequencing. BMC Med Genomics. 2018;11(1):6.
292	Roberts JL, Hovanes K, Dasouki M, Manzardo AM, Butler MG. Chromosomal microarray analysis of consecutive individuals with autism spectrum disorders or learning disability presenting for genetic services. Gene. 2014;535(1):70-8.
293	Rochtus A, Olson HE, Smith L, Keith LG, El Achkar C, Taylor A, et al. Genetic diagnoses in epilepsy: The impact of dynamic exome analysis in a pediatric cohort. Epilepsia. 2020;61(2):249-58.
294	Rodríguez-Revenga L, Vallespín E, Madrigal I, Palomares M, Mur A, García-Miñaur S, et al. A parallel study of different array-CGH platforms in a set of Spanish patients with developmental delay and intellectual disability. Gene. 2013;521(1):82-6.
295	Roselló M, Martínez F, Monfort S, Mayo S, Oltra S, Orellana C. Phenotype profiling of patients with intellectual disability and copy number variations. Eur J Paediatr Neurol. 2014;18(5):558-66.
296	Rosenfeld JA, Ballif BC, Torchia BS, Sahoo T, Ravnan JB, Schultz R, et al. Copy number variations associated with autism spectrum disorders contribute to a spectrum of neurodevelopmental disorders. Genet Med. 2010;12(11):694-702.
297	Rosina E, Pezzani L, Apuril E, Pezzoli L, Marchetti D, Bellini M, et al. Comparison of first-tier whole-exome sequencing with a multi-step traditional approach for diagnosing paediatric outpatients: An Italian prospective study. Mol Genet Genomic Med. 2023:e2316.
298	Rossi M, El-Khechen D, Black MH, Farwell Hagman KD, Tang S, Powis Z. Outcomes of Diagnostic Exome Sequencing in Patients With Diagnosed or Suspected Autism Spectrum Disorders. Pediatr Neurol. 2017;70:34-43.e2.
299	Routier L, Verny F, Barcia G, Chemaly N, Desguerre I, Colleaux L, et al. Exome sequencing findings in 27 patients with myoclonic-atonic epilepsy: Is there a major genetic factor? Clin Genet. 2019;96(3):254-60.
300	Rump P, Jazayeri O, van Dijk-Bos KK, Johansson LF, van Essen AJ, Verheij JB, et al. Whole-exome sequencing is a powerful approach for establishing the etiological diagnosis in patients with intellectual disability and microcephaly. BMC Med Genomics. 2016;9:7.
301	Sagoo GS, Mohammed S, Barton G, Norbury G, Ahn JW, Ogilvie CM, et al. Cost Effectiveness of Using Array-CGH for Diagnosing Learning Disability. Appl Health Econ Health Policy. 2015;13(4):421-32.
302	Salinas V, Martínez N, Maturo JP, Rodriguez-Quiroga SA, Zavala L, Medina N, et al. Clinical next generation sequencing in developmental and epileptic encephalopathies: Diagnostic relevance of data re-analysis and variants re-interpretation. Eur J Med Genet. 2021;64(12):104363.
303	Sandal S, Verma IC, Mahay SB, Dubey S, Sabharwal RK, Kulshrestha S, et al. Next-Generation Sequencing in Unexplained Intellectual Disability. Indian J Pediatr. 2024;91(7):682-95.
304	Sanders M, Lemmens CMC, Jansen FE, Brilstra EH, Koeleman BPC, Braun KPJ. Implications of genetic diagnostics in epilepsy surgery candidates: A single-center cohort study. Epilepsia Open.

	2019;4(4):609-17.
305	Sandoval-Talamantes AK, Mori M, Santos-Simarro F, García-Miñaur S, Mansilla E, Tenorio JA, et al. Chromosomal Microarray in Patients with Non-Syndromic Autism Spectrum Disorders in the Clinical Routine of a Tertiary Hospital. Genes (Basel). 2023;14(4).
306	Sandoval-Talamantes AK, Tenorio-Castaño JA, Santos-Simarro F, Adán C, Fernández-Elvira M, García-Fernández L, et al. NGS Custom Panel Implementation in Patients with Non-Syndromic Autism Spectrum Disorders in the Clinical Routine of a Tertiary Hospital. Genes (Basel). 2023;14(11).
307	Sansović I, Ivankov AM, Bobinec A, Kero M, Barišić I. Chromosomal microarray in clinical diagnosis: a study of 337 patients with congenital anomalies and developmental delays or intellectual disability. Croat Med J. 2017;58(3):231-8.
308	Schaefer GB, Starr L, Pickering D, Skar G, Dehaai K, Sanger WG. Array comparative genomic hybridization findings in a cohort referred for an autism evaluation. J Child Neurol. 2010;25(12):1498-503.
309	Schmidt A, Danyel M, Grundmann K, Brunet T, Klinkhammer H, Hsieh TC, et al. Next-generation phenotyping integrated in a national framework for patients with ultrarare disorders improves genetic diagnostics and yields new molecular findings. Nat Genet. 2024;56(8):1644-53.
310	Sedlackova L, Sterbova K, Vlckova M, Seeman P, Zarubova J, Marusic P, et al. Yield of exome sequencing in patients with developmental and epileptic encephalopathies and inconclusive targeted gene panel. Eur J Paediatr Neurol. 2023;48:17-29.
311	Segal E, Pedro H, Valdez-Gonzalez K, Parisotto S, Gliksman F, Thompson S, et al. Diagnostic Yield of Epilepsy Panels in Children With Medication-Refractory Epilepsy. Pediatr Neurol. 2016;64:66-71.
312	Seo GH, Lee H, Lee J, Han H, Cho YK, Kim M, et al. Diagnostic performance of automated, streamlined, daily updated exome analysis in patients with neurodevelopmental delay. Mol Med. 2022;28(1):38.
313	Sharma P, Gupta N, Chowdhury MR, Sapra S, Ghosh M, Gulati S, et al. Application of chromosomal microarrays in the evaluation of intellectual disability/global developmental delay patients - A study from a tertiary care genetic centre in India. Gene. 2016;590(1):109-19.
314	Shchubelka K, Turova L, Wolfsberger W, Kalanquin K, Williston K, Kurutsa O, et al. Genetic determinants of global developmental delay and intellectual disability in Ukrainian children. J Neurodev Disord. 2024;16(1):13.
315	Shellhaas RA, Wusthoff CJ, Tsuchida TN, Glass HC, Chu CJ, Massey SL, et al. Profile of neonatal epilepsies: Characteristics of a prospective US cohort. Neurology. 2017;89(9):893-9.
316	Shen Y, Dies KA, Holm IA, Bridgemohan C, Sobeih MM, Caronna EB, et al. Clinical genetic testing for patients with autism spectrum disorders. Pediatrics. 2010;125(4):e727-35.
317	Sheth F, Shah J, Jain D, Shah S, Patel H, Patel K, et al. Comparative yield of molecular diagnostic algorithms for autism spectrum disorder diagnosis in India: evidence supporting whole exome sequencing as first tier test. BMC Neurol. 2023;23(1):292.
318	Shevell MI, Bejjani BA, Srour M, Rorem EA, Hall N, Shaffer LG. Array comparative genomic hybridization in global developmental delay. Am J Med Genet B Neuropsychiatr Genet. 2008;147b(7):1101-8.

319	Shin S, Lee J, Kim YG, Ha C, Park JH, Kim JW, et al. Genetic Diagnosis of Children With Neurodevelopmental Disorders Using Whole Genome Sequencing. Pediatr Neurol. 2023;149:44-52.
320	Shin S, Yu N, Choi JR, Jeong S, Lee KA. Routine chromosomal microarray analysis is necessary in Korean patients with unexplained developmental delay/mental retardation/autism spectrum disorder. Ann Lab Med. 2015;35(5):510-8.
321	Shoukier M, Klein N, Auber B, Wickert J, Schröder J, Zoll B, et al. Array CGH in patients with developmental delay or intellectual disability: are there phenotypic clues to pathogenic copy number variants? Clin Genet. 2013;83(1):53-65.
322	Siu WK, Lam CW, Mak CM, Lau ET, Tang MH, Tang WF, et al. Diagnostic yield of array CGH in patients with autism spectrum disorder in Hong Kong. Clin Transl Med. 2016;5(1):18.
323	Snoeijen-Schouwenaars FM, van Ool JS, Verhoeven JS, van Mierlo P, Braakman HMH, Smeets EE, et al. Diagnostic exome sequencing in 100 consecutive patients with both epilepsy and intellectual disability. Epilepsia. 2019;60(1):155-64.
324	Soden SE, Saunders CJ, Willig LK, Farrow EG, Smith LD, Petrikin JE, et al. Effectiveness of exome and genome sequencing guided by acuity of illness for diagnosis of neurodevelopmental disorders. Sci Transl Med. 2014;6(265):265ra168.
325	Srivastava S, Cohen JS, Vernon H, Barañano K, McClellan R, Jamal L, et al. Clinical whole exome sequencing in child neurology practice. Ann Neurol. 2014;76(4):473-83.
326	Staněk D, Laššuthová P, Štěrbová K, Vlčková M, Neupauerová J, Krůtová M, et al. Detection rate of causal variants in severe childhood epilepsy is highest in patients with seizure onset within the first four weeks of life. Orphanet J Rare Dis. 2018;13(1):71.
327	Stavropoulos DJ, Merico D, Jobling R, Bowdin S, Monfared N, Thiruvahindrapuram B, et al. Whole Genome Sequencing Expands Diagnostic Utility and Improves Clinical Management in Pediatric Medicine. NPJ Genom Med. 2016;1:15012-.
328	Stobbe G, Liu Y, Wu R, Hudgings LH, Thompson O, Hisama FM. Diagnostic yield of array comparative genomic hybridization in adults with autism spectrum disorders. Genet Med. 2014;16(1):70-7.
329	Stödberg T, Tomson T, Barbaro M, Stranneheim H, Anderlid BM, Carlsson S, et al. Epilepsy syndromes, etiologies, and the use of next-generation sequencing in epilepsy presenting in the first 2 years of life: A population-based study. Epilepsia. 2020;61(11):2486-99.
330	Stojanovic JR, Miletic A, Peterlin B, Maver A, Mijovic M, Borlja N, et al. Diagnostic and Clinical Utility of Clinical Exome Sequencing in Children With Moderate and Severe Global Developmental Delay / Intellectual Disability. J Child Neurol. 2020;35(2):116-31.
331	Stranneheim H, Lagerstedt-Robinson K, Magnusson M, Kvarnung M, Nilsson D, Lesko N, et al. Integration of whole genome sequencing into a healthcare setting: high diagnostic rates across multiple clinical entities in 3219 rare disease patients. Genome Med. 2021;13(1):40.
332	Streață I, Caramizaru A, Riza AL, Șerban-Sosoi S, Pîrvu A, Cara ML, et al. Pathogenic Copy Number Variations Involved in the Genetic Etiology of Syndromic and Non-Syndromic Intellectual Disability-Data from a Romanian Cohort. Diagnostics (Basel). 2022;12(12).
333	Su M, Page S, Haag M, Swisshelm K, Hennerich D, Graw S, et al. Clinical utility and cost-effectiveness analysis of chromosome testing concomitant with chromosomal microarray of

	patients with constitutional disorders in a U.S. academic medical center. J Genet Couns. 2022;31(2):364-74.
334	Suga K, Imoto I, Ito H, Naruto T, Goji A, Osumi K, et al. Next-generation sequencing for the diagnosis of patients with congenital multiple anomalies and / or intellectual disabilities. J Med Invest. 2020;67(3.4):246-9.
335	Sun D, Liu Y, Cai W, Ma J, Ni K, Chen M, et al. Detection of Disease-Causing SNVs/Indels and CNVs in Single Test Based on Whole Exome Sequencing: A Retrospective Case Study in Epileptic Encephalopathies. Front Pediatr. 2021;9:635703.
336	Sun Y, Peng J, Liang D, Ye X, Xu N, Chen L, et al. Genome sequencing demonstrates high diagnostic yield in children with undiagnosed global developmental delay/intellectual disability: A prospective study. Hum Mutat. 2022;43(5):568-81.
337	Symonds JD, Zuberi SM, Stewart K, McLellan A, O'Regan M, MacLeod S, et al. Incidence and phenotypes of childhood-onset genetic epilepsies: a prospective population-based national cohort. Brain. 2019;142(8):2303-18.
338	Sys M, van den Bogaert A, Roosens B, Lampo A, Jansen A, Wouters S, et al. Can clinical characteristics be criteria to perform chromosomal microarray analysis in children and adolescents with autism spectrum disorders? Minerva Pediatr. 2018;70(3):225-32.
339	Tal-Ben Ishay R, Shil A, Solomon S, Sadigurschi N, Abu-Kaf H, Meiri G, et al. Diagnostic Yield and Economic Implications of Whole-Exome Sequencing for ASD Diagnosis in Israel. Genes (Basel). 2021;13(1).
340	Tammimies K, Marshall CR, Walker S, Kaur G, Thiruvahindrapuram B, Lionel AC, et al. Molecular Diagnostic Yield of Chromosomal Microarray Analysis and Whole-Exome Sequencing in Children With Autism Spectrum Disorder. Jama. 2015;314(9):895-903.
341	Tan CA, Topper S, Del Gaudio D, Nelakuditi V, Shchelochkov O, Nowaczyk MJM, et al. Characterization of patients referred for non-specific intellectual disability testing: the importance of autosomal genes for diagnosis. Clin Genet. 2016;89(4):478-83.
342	Tao VQ, Chan KY, Chu YW, Mok GT, Tan TY, Yang W, et al. The clinical impact of chromosomal microarray on paediatric care in Hong Kong. PLoS One. 2014;9(10):e109629.
343	Tarailo-Graovac M, Shyr C, Ross CJ, Horvath GA, Salvarinova R, Ye XC, et al. Exome Sequencing and the Management of Neurometabolic Disorders. N Engl J Med. 2016;374(23):2246-55.
344	Taşkıran EZ, Karaosmanoğlu B, Koşukcu C, Ürel-Demir G, Akgün-Doğan Ö, Şimşek-Kiper P, et al. Diagnostic yield of whole-exome sequencing in non-syndromic intellectual disability. J Intellect Disabil Res. 2021;65(6):577-88.
345	Taylor MR, Jirikowic J, Wells C, Springer M, McGavran L, Lunt B, et al. High prevalence of array comparative genomic hybridization abnormalities in adults with unexplained intellectual disability. Genet Med. 2010;12(1):32-8.
346	Thevenon J, Duffourd Y, Masurel-Paulet A, Lefebvre M, Feillet F, El Chehadeh-Djebbar S, et al. Diagnostic odyssey in severe neurodevelopmental disorders: toward clinical whole-exome sequencing as a first-line diagnostic test. Clin Genet. 2016;89(6):700-7.
347	Tong W, Wang Y, Lu Y, Ye T, Song C, Xu Y, et al. Whole-exome Sequencing Helps the Diagnosis and Treatment in Children with Neurodevelopmental Delay Accompanied Unexplained Dyspnea. Sci Rep. 2018;8(1):5214.

348	Trakadis Y, Accogli A, Qi B, Bloom D, Joober R, Levy E, et al. Next-generation gene panel testing in adolescents and adults in a medical neuropsychiatric genetics clinic. Neurogenetics. 2021;22(4):313-22.
349	Tran KT, Le VS, Bui HTP, Do DH, Ly HTT, Nguyen HT, et al. Genetic landscape of autism spectrum disorder in Vietnamese children. Sci Rep. 2020;10(1):5034.
350	Tran Mau-Them F, Moutton S, Racine C, Vitobello A, Bruel AL, Nambot S, et al. Second-tier trio exome sequencing after negative solo clinical exome sequencing: an efficient strategy to increase diagnostic yield and decipher molecular bases in undiagnosed developmental disorders. Hum Genet. 2020;139(11):1381-90.
351	Triono A, Iskandar K, Hadiyanto ML, Nugrahanto AP, Diantika K, Wijayanti VW, et al. Identification of the genetic basis of pediatric neurogenetic disorders at a tertiary referral hospital in Indonesia: Contribution of whole exome sequencing. PLoS One. 2023;18(10):e0293113.
352	Trump N, McTague A, Brittain H, Papandreou A, Meyer E, Ngoh A, et al. Improving diagnosis and broadening the phenotypes in early-onset seizure and severe developmental delay disorders through gene panel analysis. J Med Genet. 2016;53(5):310-7.
353	Truty R, Patil N, Sankar R, Sullivan J, Millichap J, Carvill G, et al. Possible precision medicine implications from genetic testing using combined detection of sequence and intragenic copy number variants in a large cohort with childhood epilepsy. Epilepsia Open. 2019;4(3):397-408.
354	Tsai MH, Chan CK, Chang YC, Lin CH, Liou CW, Chang WN, et al. Molecular Genetic Characterization of Patients With Focal Epilepsy Using a Customized Targeted Resequencing Gene Panel. Front Neurol. 2018;9:515.
355	Tsang MH, Leung GK, Ho AC, Yeung KS, Mak CC, Pei SL, et al. Exome sequencing identifies molecular diagnosis in children with drug-resistant epilepsy. Epilepsia Open. 2019;4(1):63-72.
356	Tsuchida N, Nakashima M, Kato M, Heyman E, Inui T, Haginoya K, et al. Detection of copy number variations in epilepsy using exome data. Clin Genet. 2018;93(3):577-87.
357	Tucker T, Montpetit A, Chai D, Chan S, Chénier S, Coe BP, et al. Comparison of genome-wide array genomic hybridization platforms for the detection of copy number variants in idiopathic mental retardation. BMC Med Genomics. 2011;4:25.
358	Tumienė B, Maver A, Writzl K, Hodžić A, Čuturilo G, Kuzmanić-Šamija R, et al. Diagnostic exome sequencing of syndromic epilepsy patients in clinical practice. Clin Genet. 2018;93(5):1057-62.
359	Turkdogan D, Turkyilmaz A, Sager G, Ozturk G, Unver O, Say M. Chromosomal microarray and exome sequencing in unexplained early infantile epileptic encephalopathies in a highly consanguineous population. Int J Neurosci. 2021:1-18.
360	Türkyılmaz A, Geckinli BB, Tekin E, Ates EA, Yarali O, Cebi AH, et al. Array-Based Comparative Genomic Hybridization Analysis in Children with Developmental Delay/Intellectual Disability. Balkan J Med Genet. 2021;24(2):15-24.
361	Türkyılmaz A, Sağer SG, Tekin E, Teralı K, Düzkalır H, Eser M, et al. Expanding the clinical and genetic landscape of (developmental) epileptic encephalopathy with spike-and-wave activation in sleep: results from studies of a Turkish cohort. Neurogenetics. 2024.
362	Vadlamudi L, Bennett CM, Tom M, Abdulrasool G, Brion K, Lundie B, et al. A Multi-Disciplinary Team Approach to Genomic Testing for Drug-Resistant Epilepsy Patients-The GENIE Study. J Clin Med. 2022;11(14).

363	Valaparambil KA, Fasaludeen A, Priya L, Menon RN, Menon R, Sundaram S. Clinical Utility of Proband Only Clinical Exome Sequencing in Neurodevelopmental Disorders. Indian J Pediatr. 2023.
364	van Niekerk M, Moosa S, van Toorn R, Solomons R. Utility of next generation sequencing in paediatric neurological disorders: experience from South Africa. Eur J Hum Genet. 2024.
365	van Slobbe M, van Haeringen A, Vissers L, Bijlsma EK, Rutten JW, Suerink M, et al. Reanalysis of whole-exome sequencing (WES) data of children with neurodevelopmental disorders in a standard patient care context. Eur J Pediatr. 2023.
366	Varesio C, Gana S, Asaro A, Ballante E, Cabini RF, Tartara E, et al. Diagnostic Yield and Cost-Effectiveness of "Dynamic" Exome Analysis in Epilepsy with Neurodevelopmental Disorders: A Tertiary-Center Experience in Northern Italy. Diagnostics (Basel). 2021;11(6).
367	Veltra D, Tilemis FN, Marinakis NM, Svingou M, Mitrakos A, Kosma K, et al. Combined exome analysis and exome depth assessment achieve a high diagnostic yield in an epilepsy case series, revealing significant genomic heterogeneity and novel mechanisms. Expert Rev Mol Diagn. 2023;23(1):85-103.
368	Vianna GS, Medeiros PF, Alves AF, Silva TO, Jehee FS. Array-CGH analysis in patients with intellectual disability and/or congenital malformations in Brazil. Genet Mol Res. 2016;15(1).
369	Viñas-Jornet M, Esteba-Castillo S, Baena N, Ribas-Vidal N, Ruiz A, Torrents-Rodas D, et al. High Incidence of Copy Number Variants in Adults with Intellectual Disability and Co-morbid Psychiatric Disorders. Behav Genet. 2018;48(4):323-36.
370	Vlaskamp DRM, Callenbach PMC, Rump P, Giannini LAA, Dijkhuizen T, Brouwer OF, et al. Copy number variation in a hospital-based cohort of children with epilepsy. Epilepsia Open. 2017;2(2):244-54.
371	Vrijenhoek T, Middelburg EM, Monroe GR, van Gassen KLI, Geenen JW, Hövels AM, et al. Whole-exome sequencing in intellectual disability; cost before and after a diagnosis. Eur J Hum Genet. 2018;26(11):1566-71.
372	Wang J, Gotway G, Pascual JM, Park JY. Diagnostic yield of clinical next-generation sequencing panels for epilepsy. JAMA Neurol. 2014;71(5):650-1.
373	Wang J, Wang Y, Wang L, Chen WY, Sheng M. The diagnostic yield of intellectual disability: combined whole genome low-coverage sequencing and medical exome sequencing. BMC Med Genomics. 2020;13(1):70.
374	Wang J, Wen Y, Zhang Q, Yu S, Chen Y, Wu X, et al. Gene mutational analysis in a cohort of Chinese children with unexplained epilepsy: Identification of a new KCND3 phenotype and novel genes causing Dravet syndrome. Seizure. 2019;66:26-30.
375	Wang JH, Xie H, Xu Q, Tian Y, Wang X, Shangguan SF, et al. Explore the value of whole exome sequencing in early diagnosis for children with language delay/disorder. Zhonghua yu fang yi xue za zhi [Chinese journal of preventive medicine]. 2021;55(7):827-34.
376	Wang R, Lei T, Fu F, Li R, Jing X, Yang X, et al. Application of chromosome microarray analysis in patients with unexplained developmental delay/intellectual disability in South China. Pediatr Neonatol. 2019;60(1):35-42.
377	Ware TL, Huskins SR, Grinton BE, Liu YC, Bennett MF, Harvey M, et al. Epidemiology and etiology of infantile developmental and epileptic encephalopathies in Tasmania. Epilepsia Open.

	2019;4(3):504-10.
378	Wayhelova M, Smetana J, Vallova V, Hladilkova E, Filkova H, Hanakova M, et al. The clinical benefit of array-based comparative genomic hybridization for detection of copy number variants in Czech children with intellectual disability and developmental delay. BMC Med Genomics. 2019;12(1):111.
379	Willemsen MH, Kleefstra T. Making headway with genetic diagnostics of intellectual disabilities. Clin Genet. 2014;85(2):101-10.
380	Willimsky EK, Munzig A, Mayer K, Biskup S, Abicht A, Hoertnagel K, et al. Next Generation Sequencing in Pediatric Epilepsy Using Customized Panels: Size Matters. Neuropediatrics. 2021;52(2):92-7.
381	Wincent J, Anderlid BM, Lagerberg M, Nordenskjöld M, Schoumans J. High-resolution molecular karyotyping in patients with developmental delay and/or multiple congenital anomalies in a clinical setting. Clin Genet. 2011;79(2):147-57.
382	Wincent J, Kolbjer S, Martin D, Luthman A, Åmark P, Dahlin M, et al. Copy number variations in children with brain malformations and refractory epilepsy. Am J Med Genet A. 2015;167a(3):512-23.
383	Wirrell EC, Shellhaas RA, Joshi C, Keator C, Kumar S, Mitchell WG. How should children with West syndrome be efficiently and accurately investigated? Results from the National Infantile Spasms Consortium. Epilepsia. 2015;56(4):617-25.
384	Wiśniowiecka-Kowalnik B, Kastory-Bronowska M, Bartnik M, Derwińska K, Dymczak-Domini W, Szumbarska D, et al. Application of custom-designed oligonucleotide array CGH in 145 patients with autistic spectrum disorders. Eur J Hum Genet. 2013;21(6):620-5.
385	Witzel MGW, Gebhard C, Wenzel S, Kleier S, Eichhorn B, Lorenz P, et al. Prospective evaluation of NGS-based sequencing in epilepsy patients: results of seven NASGE-associated diagnostic laboratories. Front Neurol. 2023;14:1276238.
386	Wojcik MH, Lemire G, Berger E, Zaki MS, Wissmann M, Win W, et al. Genome Sequencing for Diagnosing Rare Diseases. N Engl J Med. 2024;390(21):1985-97.
387	Wolfe K, Strydom A, Morrogh D, Carter J, Cutajar P, Eyeoyibo M, et al. Chromosomal microarray testing in adults with intellectual disability presenting with comorbid psychiatric disorders. Eur J Hum Genet. 2016;25(1):66-72.
388	Won D, Kim SH, Kim B, Lee ST, Kang HC, Choi JR. Reanalysis of Genomic Sequencing Results in a Clinical Laboratory: Advantages and Limitations. Front Neurol. 2020;11:612.
389	Wu CC, Tsai MH, Chu YJ, Weng WC, Fan PC, Lee WT. The role of targeted gene panel in pediatric drug-resistant epilepsy. Epilepsy Behav. 2020;106:107003.
390	Wu D, Wu Y, Lan Y, Lan S, Zhong Z, Li D, et al. Chromosomal Aberrations in Pediatric Patients With Moderate/Severe Developmental Delay/Intellectual Disability With Abundant Phenotypic Heterogeneities: A Single-Center Study. Pediatr Neurol. 2023;147:72-81.
391	Wu HR, Li L, Ma YN, Liu CL, Pei P, Zheng XF, et al. [The diagnostic value of chromosome microarray analysis technique in the genetic causes of children with intellectual disability or global developmental delay]. Zhonghua Yi Xue Za Zhi. 2021;101(3):224-8.
392	Wu R, Li X, Meng Z, Li P, He Z, Liang L. Phenotypic and genetic analysis of children with unexplained neurodevelopmental delay and neurodevelopmental comorbidities in a Chinese cohort

	using trio-based whole-exome sequencing. Orphanet J Rare Dis. 2024;19(1):205.
393	Xiang B, Zhu H, Shen Y, Miller DT, Lu K, Hu X, et al. Genome-wide oligonucleotide array comparative genomic hybridization for etiological diagnosis of mental retardation: a multicenter experience of 1499 clinical cases. J Mol Diagn. 2010;12(2):204-12.
394	Xiang J, Ding Y, Yang F, Gao A, Zhang W, Tang H, et al. Genetic Analysis of Children With Unexplained Developmental Delay and/or Intellectual Disability by Whole-Exome Sequencing. Front Genet. 2021;12:738561.
395	Xiao B, Qiu W, Ji X, Liu X, Huang Z, Liu H, et al. Marked yield of re-evaluating phenotype and exome/target sequencing data in 33 individuals with intellectual disabilities. Am J Med Genet A. 2018;176(1):107-15.
396	Xu M, Ji Y, Zhang T, Jiang X, Fan Y, Geng J, et al. Clinical Application of Chromosome Microarray Analysis in Han Chinese Children with Neurodevelopmental Disorders. Neurosci Bull. 2018;34(6):981-91.
397	Xu Q, Goldstein J, Wang P, Gadi IK, Labreche H, Rehder C, et al. Chromosomal microarray analysis in clinical evaluation of neurodevelopmental disorders-reporting a novel deletion of SETDB1 and illustration of counseling challenge. Pediatr Res. 2016;80(3):371-81.
398	Yamamoto T, Imaizumi T, Yamamoto-Shimojima K, Lu Y, Yanagishita T, Shimada S, et al. Genomic backgrounds of Japanese patients with undiagnosed neurodevelopmental disorders. Brain Dev. 2019;41(9):776-82.
399	Yan H, Shi Z, Wu Y, Xiao J, Gu Q, Yang Y, et al. Targeted next generation sequencing in 112 Chinese patients with intellectual disability/developmental delay: novel mutations and candidate gene. BMC Med Genet. 2019;20(1):80.
400	Yang EH, Shin YB, Choi SH, Yoo HW, Kim HY, Kwak MJ, et al. Chromosomal Microarray in Children With Developmental Delay: The Experience of a Tertiary Center in Korea. Front Pediatr. 2021;9:690493.
401	Yang L, Kong Y, Dong X, Hu L, Lin Y, Chen X, et al. Clinical and genetic spectrum of a large cohort of children with epilepsy in China. Genet Med. 2019;21(3):564-71.
402	Yang M, Xu B, Wang J, Zhang Z, Xie H, Wang H, et al. Genetic diagnoses in pediatric patients with epilepsy and comorbid intellectual disability. Epilepsy Res. 2021;170:106552.
403	Yuan H, Shangguan S, Li Z, Luo J, Su J, Yao R, et al. CNV profiles of Chinese pediatric patients with developmental disorders. Genet Med. 2021;23(4):669-78.
404	Yüksel Ülker A, Uludağ Alkaya D, Çağlayan AO, Usluer E, Aykut A, Aslanger A, et al. An investigation of the etiology and follow-up findings in 35 children with overgrowth syndromes, including biallelic SUZ12 variant. Am J Med Genet A. 2023;191(6):1530-45.
405	Zacher P, Mayer T, Brandhoff F, Bartolomaeus T, Le Duc D, Finzel M, et al. The genetic landscape of intellectual disability and epilepsy in adults and the elderly: a systematic genetic work-up of 150 individuals. Genet Med. 2021;23(8):1492-7.
406	Zaganas I, Vorgia P, Spilioti M, Mathioudakis L, Raissaki M, Ilia S, et al. Genetic cause of epilepsy in a Greek cohort of children and young adults with heterogeneous epilepsy syndromes. Epilepsy Behav Rep. 2021;16:100477.
407	Zhai Y, Zhang Z, Shi P, Martin DM, Kong X. Incorporation of exome-based CNV analysis makes trio-WES a more powerful tool for clinical diagnosis in neurodevelopmental disorders: A

	retrospective study. Hum Mutat. 2021;42(8):990-1004.
408	Zhang H, Chen X, Tan H, Teng Y, Liu D, Wu J, et al. The exploration of genetic aetiology and diagnostic strategy for 321 Chinese individuals with intellectual disability. Clin Chim Acta. 2023;538:94-103.
409	Zhang J, Xu Y, Liu Y, Yue L, Jin H, Chen Y, et al. Genetic Testing for Global Developmental Delay in Early Childhood. JAMA Netw Open. 2024;7(6):e2415084.
410	Zhang L, Gao J, Liu H, Tian Y, Zhang X, Lei W, et al. Pathogenic variants identified by whole-exome sequencing in 43 patients with epilepsy. Hum Genomics. 2020;14(1):44.
411	Zhang Q, Li J, Zhao Y, Bao X, Wei L, Wang J. Gene mutation analysis of 175 Chinese patients with early-onset epileptic encephalopathy. Clin Genet. 2017;91(5):717-24.
412	Zhang Y, Kong W, Gao Y, Liu X, Gao K, Xie H, et al. Gene Mutation Analysis in 253 Chinese Children with Unexplained Epilepsy and Intellectual/Developmental Disabilities. PLoS One. 2015;10(11):e0141782.
413	Zhang Y, Li Y, Guo R, Xu W, Liu X, Zhao C, et al. Genetic diagnostic yields of 354 Chinese ASD children with rare mutations by a pipeline of genomic tests. Front Genet. 2023;14:1108440.
414	Zhou P, He N, Zhang JW, Lin ZJ, Wang J, Yan LM, et al. Novel mutations and phenotypes of epilepsy-associated genes in epileptic encephalopathies. Genes Brain Behav. 2018;17(8):e12456.
415	Zhou WZ, Zhang J, Li Z, Lin X, Li J, Wang S, et al. Targeted resequencing of 358 candidate genes for autism spectrum disorder in a Chinese cohort reveals diagnostic potential and genotype-phenotype correlations. Hum Mutat. 2019;40(6):801-15.
416	Zou D, Wang L, Liao J, Xiao H, Duan J, Zhang T, et al. Genome sequencing of 320 Chinese children with epilepsy: a clinical and molecular study. Brain. 2021;144(12):3623-34.

Supplementary Table 3. Newcastle-Ottawa Scale (NOS) Quality Assessment

study	Representativeness of the sample	Sample size	Non-respondents	Ascertainment of the exposure	Comparability	Assessment of outcome	Statistical test	Total
Abdi 2023	1	1	0	2	1	2	1	8
Abe-Hatano 2021	1	0	0	2	1	2	1	7
Agarwala 2023	1	0	0	2	2	2	1	8
Akter 2023	1	1	0	2	1	2	1	8
Al Anazi 2022	1	0	0	2	1	2	1	7
Albuz 2021	1	0	0	2	1	2	1	7
Ali 2019	1	0	0	2	1	2	1	7
Al-Kasbi 2022	1	0	0	2	1	2	1	7
Allen 2015	1	0	0	2	1	2	1	7
Allen 2016	1	0	0	2	1	2	1	7
Allen 2021	1	0	0	2	1	2	1	7
Al-Mamari 2023	1	0	0	2	2	2	1	8
Al-Nabhani 2018	1	0	0	2	1	2	1	7
Alotibi 2023	1	0	0	2	1	2	1	7
Al-Sarraj 2024	1	0	0	2	1	2	1	7
Alsubaie 2020	1	1	0	2	1	2	1	8
Alvarez-Mora 2016	1	0	0	2	1	2	1	7
Alvarez-Mora 2023	1	1	0	2	1	2	1	8
Amado-Puentes 2019	1	1	0	2	2	2	1	9
Amadori 2020	1	0	0	2	1	2	1	7

Angione 2019	1	0	0	2	1	2	1	7
Annunziata 2023	1	1	0	2	1	2	1	8
Arafat 2017	1	0	0	2	1	2	1	7
Arican 2019	1	0	0	2	1	2	1	7
Arnaud 2022	1	1	0	2	1	2	1	8
Aspromonte 2019	1	0	0	2	1	2	1	7
Atli 2022	1	0	0	2	1	2	1	7
Baccarin 2020	1	0	0	2	1	2	1	7
Badura-Stronka 2023	1	0	0	2	1	2	1	7
Baer 2024	1	1	0	2	1	2	1	8
Bakshi 2020	1	0	0	2	1	2	1	7
Balciuniene 2019	1	0	0	2	1	2	1	7
Balicza 2019	1	0	0	2	1	2	1	7
Baris 2007	1	1	0	2	1	2	1	8
Baris 2023	1	0	0	2	1	2	1	7
Bartnik 2012	1	0	0	2	1	2	1	7
Bartnik 2014	1	1	0	2	1	2	1	8
Bartnik 2014	1	1	0	2	1	2	1	8
Bashiri 2023	1	0	0	2	1	2	1	7
Battaglia 2013	1	0	0	2	1	2	1	7
Bayanova 2023	1	0	0	2	1	2	1	7
Bayat 2022	1	1	1	2	1	2	1	9

Becker 2024	1	0	0	2	1	2	1	7
Behjati 2013	1	0	0	2	1	2	1	7
Benson 2020	1	0	0	2	1	2	1	7
Berg 2017	1	1	1	2	2	2	1	10
Blazekovic 2022	1	1	0	2	1	2	1	8
Boggula 2015	1	0	0	2	1	2	1	7
Boonsimma 2023	1	0	0	2	1	2	1	7
Borlot 2019	1	0	0	2	1	2	1	7
Boutry-Kryza 2015	1	0	0	2	1	2	1	7
Bruno 2021	1	0	0	2	1	2	1	7
Bui 2024	1	0	0	2	1	2	1	7
Burk 2024	1	1	0	2	2	2	1	9
Butler 2017	1	1	0	2	1	2	1	8
Byeon 2014	1	0	0	2	1	2	1	7
Calderoni 2020	1	0	0	2	1	2	1	7
Canafoglia 2021	1	0	0	2	1	2	1	7
Capkova 2019	1	0	0	2	1	2	1	7
Cappuccio 2016	1	1	0	2	2	2	1	9
Caramaschi 2014	1	0	0	2	2	2	1	8
Castellotti 2024	1	1	1	2	2	2	1	10
Castells-Sarret 2018	1	1	0	2	1	2	1	8
Catusi 2020	1	1	0	2	2	2	1	9

Çebi 2020	1	1	0	2	1	2	1	8
Chan 2018	1	1	0	2	2	2	1	9
Chaves 2019	1	1	0	2	1	2	1	8
Chehbani 2022	1	0	0	2	2	2	1	8
Chen 2021	1	1	0	2	1	2	1	8
Chengyan 2024	1	0	0	2	1	2	1	7
Chuan 2022	1	0	0	2	1	2	1	7
Coppola 2019	1	1	0	2	2	2	1	9
Costa 2022	1	0	0	2	1	2	1	7
Costain 2019	1	0	1	2	1	2	1	8
Coutton 2015	1	0	0	2	1	2	1	7
Cucinotta 2023	1	1	0	2	1	2	1	8
D’ Arrigo 2015	1	0	0	2	1	2	1	7
D’ Gama 2023	1	0	1	2	1	2	1	8
da Cunha Leite 2022	1	1	1	2	1	2	1	9
Dai 2021	1	1	0	2	1	2	1	8
D’ Amours 2014	1	0	0	2	1	2	1	7
de Ligt 2012	1	0	0	2	1	2	1	7
de Souza 2019	1	0	0	2	1	2	1	7
De Wachter 2024	1	1	0	2	2	2	1	9
Della Mina 2015	1	0	0	2	1	2	1	7
Demarest 2022	1	0	0	2	1	2	1	7

Demos 2019	1	0	0	2	1	2	1	7
Di Gregorio 2017	1	1	0	2	2	2	1	9
Dimassi 2016	1	0	0	2	1	2	1	7
Dong 2020	1	1	0	2	1	2	1	8
Du 2014	1	0	0	2	1	2	1	7
Du 2018	1	0	0	2	1	2	1	7
Duc 2023	1	0	0	2	1	2	1	7
Epilepsy Genetics Initiative, 2019	1	0	0	2	1	2	1	7
Espeche 2020	1	0	0	2	1	2	1	7
Essajee 2022	1	0	0	2	1	2	1	7
Evers 2017	1	0	0	2	1	2	1	7
Ewams 2018	1	0	0	2	1	2	1	7
Fan 2018	1	1	0	2	1	2	1	8
Fan 2018	1	1	0	2	1	2	1	8
Fernắndez-Marmiesse 2019	1	1	0	2	1	2	1	8
Firouzabadi 2017	1	0	0	2	1	2	1	7
Francis 2023	1	1	0	2	1	2	1	8
Fung 2017	1	0	0	2	1	2	1	7
Galizia 2012	1	0	0	2	1	2	1	7
Gall 2021	1	1	0	2	1	2	1	8

Gao 2019	1	0	0	2	1	2	1	7
Garrido-Torres 2024	1	1	0	2	2	2	1	9
Gerik-Celebi 2023	1	0	0	2	1	2	1	7
Gieldon 2018	1	0	0	2	1	2	1	7
Gilissen 2014	1	0	0	2	1	2	1	7
Gökben 2016	1	0	0	2	1	2	1	7
Graifman 2023	1	0	0	2	1	2	1	7
Grether 2023	1	0	0	2	1	2	1	7
Grozeva 2015	1	1	0	2	1	2	1	8
Guo 2021	1	1	0	2	1	2	1	8
Halvardson 2016	1	0	0	2	1	2	1	7
Hamdan 2017	1	0	0	2	1	2	1	7
Han 2018	1	0	0	2	1	2	1	7
Han 2019	1	0	0	2	1	2	1	7
Havilang 2023	1	1	0	2	2	2	1	9
Helbig 2016	1	1	0	2	2	2	1	9
Hesse 2018	1	1	0	2	1	2	1	8
Hildebrand 2016	1	1	0	2	1	2	1	8
Hino-Fukuyo 2015	1	0	0	2	1	2	1	7
Hiraide 2021	1	0	0	2	1	2	1	7
Hiz Kurul 2022	1	1	0	2	1	2	1	8
Hnoonual 2017	1	0	0	2	1	2	1	7

Ho 2016	1	1	0	2	1	2	1	8
Ho 2016	1	1	0	2	1	2	1	8
Hoelz 2020	1	0	0	2	1	2	1	7
Hoelz 2020	1	0	0	2	1	2	1	7
Horák 2023	1	0	0	2	1	2	1	7
Howell 2013	1	1	0	2	1	2	1	8
Howell 2018	1	0	0	2	1	2	1	7
Hsu 2023	1	0	0	2	1	2	1	7
Hu 2019	1	1	0	2	1	2	1	8
Hu 2023	1	0	0	2	1	2	1	7
Ibarluzea 2020	1	0	0	2	1	2	1	7
Iourov 2012	1	0	0	2	1	2	1	7
Ishay 2022	1	0	0	2	1	2	1	7
Jalkh 2019	1	1	0	2	1	2	1	8
Jang 2019	1	1	0	2	1	2	1	8
Jang 2019	1	0	0	2	1	2	1	7
Jezela Stanek 2020	1	0	0	2	1	2	1	7
Jiang 2020	1	1	0	2	1	2	1	8
Jiao 2019	1	1	1	2	1	2	1	9
João 2024	1	0	0	2	1	2	1	7
Johannesen 2020	1	0	0	2	1	2	1	7
Joly 2001	1	0	0	2	1	2	1	7

Jose 2024	1	0	0	2	1	2	1	7
Kahrizi 2018	1	0	0	2	1	2	1	7
Kalsner 2017	1	0	0	2	1	2	1	7
Kamath 2020	1	0	0	2	1	2	1	7
Kang 2019	1	1	0	2	1	2	1	8
Kanmaz 2024	1	1	1	2	1	2	1	9
Kashevarova 2014	1	0	0	2	1	2	1	7
Kim 2019	1	0	0	2	1	2	1	7
Kim 2023	1	0	0	2	1	2	1	7
Kim 2023	1	1	0	2	2	2	1	9
Kim 2024	1	0	0	2	1	2	1	7
Kirchhoff 2004	1	0	0	2	1	2	1	7
Ko 2018	1	1	0	2	1	2	1	8
Kodera 2013	1	0	0	2	1	2	1	7
Koh 2003	1	1	0	2	2	2	1	9
Kousoulidou 2013	1	0	0	2	1	2	1	7
Krenn 2020	1	0	0	2	1	2	1	7
Krepischi 2022	1	1	0	2	1	2	1	8
Krey 2020	1	0	0	2	1	2	1	7
Krgovic 2022	1	0	0	2	1	2	1	7
Krygier 2023	1	0	0	2	1	2	1	7
Lee 2013	1	0	0	2	1	2	1	7

Lee 2017	1	0	0	2	1	2	1	7
Lee 2017	1	0	0	2	1	2	1	7
Lee 2018	1	1	0	2	1	2	1	8
Lee 2018	1	0	0	2	1	2	1	7
Lee 2019	1	0	0	2	1	2	1	7
Lee 2020	1	0	0	2	1	2	1	7
Lee 2020	1	0	0	2	1	2	1	7
Lee 2020	1	0	0	2	1	2	1	7
Lee 2020	1	0	0	2	1	2	1	7
Lee 2021	1	1	0	2	1	2	1	8
Lee 2021	1	0	0	2	1	2	1	7
Lee 2021	1	0	0	2	1	2	1	7
Lee 2024	1	0	0	2	1	2	1	7
Leite 2022	1	1	0	2	1	2	1	8
Lemke 2012	1	0	0	2	1	2	1	7
Lengyel 2022	1	0	0	2	1	2	1	7
Li 2019	1	0	0	2	1	2	1	7
Licchetta 2020	1	0	0	2	1	2	1	7
Lin 2023	1	0	0	2	1	2	1	7
Lindstrand 2019	1	1	0	2	1	2	1	8
Lindstrand 2022	1	1	0	2	1	2	1	8
Lintas 2017	1	0	0	2	1	2	1	7

Liu 2018	1	0	0	2	1	2	1	7
Liu 2021	1	0	0	2	1	2	1	7
Liu 2022	1	1	0	2	1	2	1	8
Lob 2024	1	1	0	2	1	2	1	8
Long 2019	1	0	0	2	1	2	1	7
Lopes 2019	1	1	0	2	1	2	1	8
Lovrečić 2018	1	0	0	2	1	2	1	7
Lowther 2023	1	1	0	2	1	2	1	8
Lphil 2017	1	0	0	2	1	2	1	7
Lumaka 2018	1	0	0	2	1	2	1	7
Lund 2013	1	0	0	2	1	2	1	7
Ma 2024	1	1	0	2	1	2	1	8
Mainali 2023	1	0	0	2	1	2	1	7
Maini 2018	1	1	0	2	2	2	1	9
Mak 2017	1	1	0	2	1	2	1	8
Manolakos 2010	1	0	0	2	1	2	1	7
Martinez Graner 2021	1	1	0	2	1	2	1	8
Martucci 2023	1	0	0	2	1	2	1	7
Marsi 2023	1	0	0	2	1	2	1	7
Mau Them 2020	1	0	0	2	1	2	1	7
McGrew 2012	1	0	0	2	1	2	1	7
McKnight 2022	1	1	0	2	1	2	1	8

Megahed 2016	1	0	0	2	1	2	1	7
Mellone 2022	1	1	0	2	1	2	1	8
Mercimek Mahmut 2015	1	0	0	2	1	2	1	7
Miao 2018	1	0	0	2	1	2	1	7
Michaud 2014	1	0	0	2	1	2	1	7
Milone 2021	1	1	0	2	1	2	1	8
Mina 2015	1	0	0	2	1	2	1	7
Mitani 2021	1	1	0	2	1	2	1	8
Mitta 2020	1	0	0	2	1	2	1	7
Moeschler 2009	1	0	0	2	1	2	1	7
Moirangthem 2021	1	0	0	2	1	2	1	7
Møller 2016	1	1	0	2	1	2	1	8
Monroe-2016	1	0	0	2	1	2	1	7
Mordaunt-2014	1	1	0	2	1	2	1	8
Morgan 2015	1	0	0	2	1	2	1	7
Muir 2019	1	0	0	2	1	2	1	7
Mullen-2013	1	1	0	2	1	2	1	8
Munnich-2019	1	1	0	2	1	2	1	8
Muona-2015	1	0	0	2	1	2	1	7
Musante-2022	1	0	0	2	1	2	1	7
Na 2020	1	0	0	2	1	2	1	7

Nambot-2018	1	1	0	2	1	2	1	8
Napoli-2018	1	0	0	2	1	2	1	7
Naseer-2015	1	0	0	2	1	2	1	7
Nassir 2022	1	0	0	2	1	2	1	7
Newman-2007	1	0	0	2	1	2	1	7
Nicholl2014	1	1	0	2	1	2	1	8
Numis-2022	1	0	0	2	1	2	1	7
Oates 2018	1	0	0	2	1	2	1	7
Ohashi 2021	1	0	0	2	1	2	1	7
Oikonomakis 2016	1	0	0	2	1	2	1	7
Olson 2014	1	1	0	2	1	2	1	8
Ortega Moreno 2017	1	0	0	2	1	2	1	7
Ostrander 2018	1	0	0	2	1	2	1	7
Palka Bayard de Volo 2021	1	1	0	2	1	2	1	8
Palmer 2014	1	0	0	2	1	2	1	7
Palmer 2018	1	0	0	2	1	2	1	7
Palmer 2021	1	0	0	2	1	2	1	7
Papuc 2019	1	0	0	2	1	2	1	7
Parrini 2016	1	1	0	2	1	2	1	8
Pekeles 2018	1	0	0	2	1	2	1	7
Peltekova 2021	1	0	0	2	2	2	1	8

Peng 2018	1	0	0	2	1	2	1	7
Peng 2019	1	1	0	2	1	2	1	8
Peng 2022	1	1	0	2	1	2	1	8
Pereira 2014	1	0	0	2	1	2	1	7
Pereira 2024	1	0	0	2	1	2	1	7
Perucca 2017	1	0	0	2	1	2	1	7
Peycheva 2018	1	0	0	2	1	2	1	7
Pfundt 2016	1	1	0	2	1	2	1	8
Qiao 2009	1	0	0	2	1	2	1	7
Quaio 2020	1	1	0	2	1	2	1	8
Rayabarapu 2022	1	1	0	2	1	2	1	8
Ream 2014	1	0	0	2	1	2	1	7
Redin 2014	1	0	0	2	1	2	1	7
Refeat 2023	1	1	0	2	1	2	1	8
Rim 2018	1	0	0	2	1	2	1	7
Roberts 2014	1	1	0	2	1	2	1	8
Rochtus 2020	1	0	0	2	1	2	1	7
Rodríguez-Revenga 2013	1	1	0	2	1	2	1	8
Rosello 2014	1	1	0	2	1	2	1	8
Rosenfeld 2010	1	1	0	2	1	2	1	8
Rosina 2023	1	0	0	2	1	2	1	7

Routier 2019	1	0	0	2	1	2	1	7
Rump 2016	1	0	0	2	1	2	1	7
Sagoo 2015	1	1	0	2	1	2	1	8
Sanders 2019	1	1	0	2	1	2	1	8
Sandoval Talama 2023	1	1	0	2	1	2	1	8
Sandoval Talama 2023	1	0	0	2	1	2	1	7
Schaefer 2010	1	1	0	2	1	2	1	8
Schmidt-2024	1	0	0	2	1	2	1	7
Sedlackova 2024	1	0	0	2	1	2	1	7
Segal 2016	1	0	0	2	1	2	1	7
Seo 2022	1	1	0	2	1	2	1	8
Sharma 2016	1	0	0	2	1	2	1	7
Shchubelka 2024	1	1	0	2	1	2	1	8
Shellhaas 2017	1	1	0	2	1	2	1	8
Shen 2010	1	1	0	2	1	2	1	8
Sheth 2023	1	0	0	2	1	2	1	7
Shevell 2008	1	0	1	2	1	2	1	8
Shin 2023	1	0	0	2	1	2	1	7
Shin 2015	1	0	0	2	1	2	1	7
Shoukier 2013	1	1	0	2	1	2	1	8

Siu 2016	1	0	0	2	1	2	1	7
Snoeijen-Schouw-2019	1	0	0	2	1	2	1	7
Soden 2014	1	0	0	2	1	2	1	7
Srivastava-2014	1	0	0	2	1	2	1	7
Stan ĕ k 2018	1	0	0	2	1	2	1	7
Stavropoulos-2016	1	0	1	2	1	2	1	8
Stobbe 2013	1	0	0	2	1	2	1	7
Stojanovic 2019	1	0	0	2	1	2	1	7
Stranneheim-2021	1	1	0	2	1	2	1	8
Su 2021	1	1	0	2	1	2	1	8
Suga 2020	1	0	0	2	1	2	1	7
Sun 2021	1	0	0	2	1	2	1	7
Sun 2022	1	0	0	2	1	2	1	7
Symonds 2019	1	1	1	2	1	2	1	9
Tal-Ben Ishay 2021	1	0	0	2	1	2	1	7
Tammimies 2015	1	1	1	2	1	2	1	9
Tan 2016	1	0	0	2	1	2	1	7
Tao 2014	1	1	0	2	1	2	1	8
Tarailo Graovac 2016	1	0	1	2	1	2	1	8
Taşkıran 2021	1	0	0	2	1	2	1	7
Taylor 2010	1	0	0	2	1	2	1	7

Thevenon 2016	1	0	0	2	1	2	1	7
Tong 2018	1	0	0	2	1	2	1	7
Trakadis 2021	1	0	0	2	1	2	1	7
Tran 2020	1	0	0	2	1	2	1	7
Triono 2023	1	0	0	2	1	2	1	7
Trump 2016	1	1	0	2	1	2	1	8
Truty 2019	1	1	0	2	1	2	1	8
Tsai 2018	1	1	0	2	1	2	1	8
Tsang 2019	1	0	0	2	1	2	1	7
Tsuchida 2018	1	0	0	2	1	2	1	7
Tucker 2011	1	0	0	2	1	2	1	7
Turkdogan 2021	1	0	0	2	1	2	1	7
Türkyılmaz 2024	1	0	0	2	1	2	1	7
Vadlamudi-2022	1	0	0	2	1	2	1	7
van Niekerk-2024	1	0	0	2	1	2	1	7
van Slobbe-2023	1	0	0	2	1	2	1	7
Varesio-2021	1	0	0	2	1	2	1	7
Vianna-2016	1	1	0	2	1	2	1	8
Vlaskamp 2017	1	1	0	2	1	2	1	8
Vrijenhoek-2018	1	1	0	2	1	2	1	8
Wang-2014	1	0	0	2	1	2	1	7
Wang 2019	1	1	0	2	1	2	1	8

Wang 2019	1	0	1	2	1	2	1	8
Wang 2020	1	0	0	2	1	2	1	7
Ware 2019	1	0	0	2	1	2	1	7
Wayhelova-2019	1	1	0	2	1	2	1	8
Willemsen-2014	1	1	0	2	1	2	1	8
Willimsky-2021	1	0	0	2	1	2	1	7
Wincent-2011	1	0	0	2	1	2	1	7
Wincent-2015	1	0	1	2	1	2	1	8
Wirrell-2015	1	1	0	2	1	2	1	8
Witzel-2023	1	1	0	2	1	2	1	8
Wojcik 2024	1	1	0	2	1	2	1	8
Wolfe-2016	1	1	0	2	1	2	1	8
Won 2020	1	1	0	2	1	2	1	8
Wu 2020	1	0	0	2	1	2	1	7
Wu 2023	1	0	0	2	1	2	1	7
Wu 2024	1	0	0	2	2	2	1	8
Xiang 2010	1	1	0	2	1	2	1	8
Xiang 2021	1	0	0	2	1	2	1	7
Xiao 2018	1	0	0	2	1	2	1	7
Xu 2016	1	0	1	2	1	2	1	8
Xu 2018	1	1	0	2	1	2	1	8
Yamamoto 2019	1	0	0	2	1	2	1	7

Yan 2019	1	0	0	2	1	2	1	7
Yang 2019	1	1	0	2	1	2	1	8
Yang 2021	1	0	0	2	1	2	1	7
Yang 2021	1	1	0	2	2	2	1	9
Yuan 2021	1	1	0	2	1	2	1	8
Zacher 2021	1	0	0	2	1	2	1	7
Zaganas 2021	1	0	0	2	1	2	1	7
Zaslan 2021	1	0	0	2	1	2	1	7
Zhai 2021	1	0	0	2	1	2	1	7
Zhang 2015	1	1	0	2	1	2	1	8
Zhang 2017	1	0	0	2	1	2	1	7
Zhang 2020	1	0	0	2	1	2	1	7
Zhang 2023	1	1	0	2	1	2	1	8
Zhang 2024	1	1	0	2	2	2	1	9
Zhou 2018	1	0	0	2	1	2	1	7
Zhou 2019	1	1	0	2	2	2	1	9
Zou 2021	1	1	0	2	1	2	1	8
